# 2,4-Diacetylphloroglucinol Reduces Beta-Amyloid Production and Secretion by Regulating ADAM10 and Intracellular Trafficking in Cellular and Animal Models of Alzheimer’s Disease

**DOI:** 10.3390/cells11162585

**Published:** 2022-08-19

**Authors:** Bong-Geum Jang, Boyoung Choi, Suyeon Kim, Duk-Shin Lee, Jisun Lee, Young Ho Koh, Sangmee Ahn Jo, Ji-Eun Kim, Tae-Cheon Kang, Min-Ju Kim

**Affiliations:** 1 Institute of Epilepsy Research, College of Medicine, Hallym University, Chuncheon 24252, Korea; 2 Department of Anatomy and Neurobiology, College of Medicine, Hallym University, Chuncheon 24252, Korea; 3 Division of Brain Disease Research, Department of Chronic Disease Convergence Research, Korea National Institute of Health, Cheongju 28159, Korea; 4 Department of Nanobiomedical Science & BK21 PLUS NBM Global Research Center for Regenerative Medicine, Dankook University, Cheonan 31116, Korea; 5 Department of Pharmacology, College of Pharmacy, Dankook University, Cheonan 31116, Korea

**Keywords:** Alzheimer’s disease, beta-amyloid, 2,4-diacetylphloroglucinol, alpha-secretase, intracellular trafficking

## Abstract

There is currently no effective treatment against Alzheimer’s disease (AD), although many strategies have been applied to reduce beta-amyloid (Aβ) levels. Here, we investigated 2,4-diacetylphloroglucinol (DAPG) effects on Aβ levels and mechanisms of action. DAPG was the most effective phloroglucinol derivative for reducing Aβ levels, without being toxic, in various models including HEK293 cells overexpressing Swedish mutant amyloid precursor protein (APP) (293sw), primary astrocytes isolated from APPsw/PS1dE9 transgenic mice, and after intrahippocampal injection of DAPG in APPsw/PS1dE9 transgenic mice. DAPG-mediated Aβ reduction was associated with increased soluble APPα (sAPPα) levels mediated by a disintegrin and metalloproteinase domain-containing protein 10 (ADAM10) but not ADAM17. ADAM10 inhibition in DAPG-treated cells prevented the effects on sAPPα but only partly on intracellular and secreted Aβ. To identify regulators of sAPPα and Aβ secretion, various inhibitors of intracellular trafficking were administered with DAPG. Brefeldin A (BFA) reversed DAPG-mediated changes in Aβ secretion in 293sw cells, whereas golgicide A (GCA) and BFA were effective in primary astrocytes, indicating a cell type-specific regulation of the trafficking. Moreover, GCA or BFA effects on sAPPα, but not Aβ, levels in primary astrocytes resembled those of ADAM10 inhibition, indicating at least partly independent trafficking pathways for sAPPα and Aβ. In conclusion, DAPG might be a promising drug candidate against AD regulating ADAM10 and intracellular trafficking, but optimizing DAPG ability to cross the BBB will be needed.

## 1. Introduction

Alzheimer’s disease (AD) is a neurodegenerative disorder affecting individuals over the age of 65 and characterized by a gradual memory decline and several pathological hallmarks including senile plaques and neurofibrillary tangles [1]. The main culprit for AD is beta-amyloid (Aβ), the major component of senile or amyloid plaques. Aβ is generated through the amyloidogenic processing, which is the cleavage of amyloid precursor protein (APP) by β- and γ-secretases. In contrast, Aβ production is attenuated by the non-amyloidogenic processing involving the activation of α- and γ-secretases. Additionally, β-secretase activation produces soluble APPβ (sAPPβ), C-terminal fragments β (CTFβ, i.e., C99 and C89), α-secretase generates sAPPα and CTFα (C83), and γ-secretase activation leads to the formation of intracellular domain of APP (AICD) from the CTFs [1,2]. APP processing encompasses the production of all APP fragments by all secretases, and its regulation is important, as it leads to physiological or pathological events induced not only by Aβ but also by other fragments [3]. Produced and secreted Aβ is cleared from the brain to the periphery by the low-density lipoprotein receptor-related protein family or is directly degraded by Aβ-degrading enzymes such as insulin-degrading enzyme, neprilysin, metalloproteinase-9 (MMP-9), and cathepsin B [1,4].

Currently, medications against AD are limited and include donepezil, an acetylcholinesterase (AchE) inhibitor used in mild to moderate AD, and memantine, a glutamate antagonist for moderate to severe AD [5]. However, these drugs only improve some symptoms and do not act on the targets recently identified by AD research. Many clinical trials on components inhibiting Aβ production through α-secretase activation or β- or γ-secretase inhibition or eliminating Aβ, such as target-specific antibodies, have been conducted. However, they were not successful as candidate components had critical side effects, such as cerebral microhemorrhages following the passive immunization with AN-1792 from Elan Pharmaceuticals, except Aducanumab, the first anti-Aβ therapy approved by the Food and Drug Administration (FDA) in 2021 [6,7]. Among several approaches to overcome these limitations, traditional screening and the screening of natural compounds against AD have been performed. Traditional new AD drug candidates include huperzine, a novel potent AchE inhibitor used in China, and phenserine, an inhibitor of dual AchE and APP [8]. Moreover, many natural compounds have shown some effectiveness in AD experimental models [9]. For example, resveratrol, epigallocatechin-3-gallate, and curcumin reduce Aβ in the cerebral cortex.

A structurally simple derivative of phloroglucinol, 2,4-diacetylphloroglucinol (DAPG), is produced from *Pseudomonas* species to protect plants against microorganisms by killing harmful prokaryotes such as fungal and protozoa involved in plant diseases such as take-all disease [10]. Besides prokaryotes, DAPG affects the plasma membrane permeability, cytosolic acidification, and reactive oxygen species (ROS) formation in *Saccharomyces cerevisiae*, a simple eukaryote. However, data regarding its effects in humans or other mammalian species or its detailed signaling mechanisms are lacking [11]. We previously reported that the butanol fraction of *Ecklonia cava*, a brown algae, reduces Aβ production, oligomer and fibril formation, and neuronal toxicity [12,13]. *Ecklonia cava* contains phlorotannin-rich components derived from phloroglucinol or 1,3,5-trihydroxybenzene, which modify chemicals produced by plants, algae, and bacteria. There are several classes, i.e., monomeric, acyl, glycoside-linked, lipid-linked, and dimer to tetrameric phlorotannins, of phloroglucinol derivatives. Their biological properties have been shown using various targets [14]. However, the compounds with a reported biological activity such as phlorotannins are mostly purified from plants or algae and, consequently, are difficult to obtain.

Here, we tested commercially available phloroglucinol derivatives to overcome this limitation and identify new active compounds as therapeutic candidates against AD. We found that DAPG significantly reduced Aβ secretion in HEK293 cells stably transfected with Swedish mutant APP (APPsw) and primary astrocytes derived from an AD transgenic mouse model. Intrahippocampal injection of DAPG in transgenic mice also reduced Triton-soluble Aβ levels, whereas intraperitoneal injections were not effective. We also examined the mechanisms triggered by DAPG to mediate Aβ decrease in the above-mentioned models. Aβ reduction was accompanied with increased intracellular and secreted sAPPα levels. DAPG effects were partly mediated by ADAM10, a potential α-secretase that decreased Aβ secretion, but not by ADAM 17. Moreover, the effects of the cotreatment with various intracellular trafficking inhibitors such as golgicide A (GCA), brefeldin A (BFA), and ZCL-278 revealed that the changes in sAPPα and Aβ secretion were mediated by intracellular trafficking pathways in a cell type-dependent manner. Therefore, although DAPG might not cross the blood–brain barrier (BBB), the present study shows the potential of DAPG or derivatives as therapeutic components against AD.

## 2. Materials and Methods

### 2.1. Materials

DAPG, phloroglucinol triacetate, 2,4,6-Trihydroxybenzaldehyde, phloroacetophenone, 2,4,6-triacetylphloroglucinol, and 2,4,6-trihydroxybenzoic acid monohydrate were obtained from Santa Cruz biotechnology (Dallas, TX, USA). Phloroglucinol, scyllo-inositol, phloretin, 1,3,5-trimethylbezene, BFA, GCA, GI254023X, TAPI, and methylthiazolyldiphenyl-tetrazolium bromide (MTT) were purchased from Sigma Aldrich (St. Louis, MO, USA). Flopropione and maclurin were purchased from Selleckchem (USA) and MP biomedicals (San Diego, CA, USA), respectively. Dulbecco’s phosphate-buffered saline (DPBS), Dulbecco’s modified Eagle’s medium (DMEM), fetal bovine serum (FBS), and 100× antibiotic-antimycotic solution were purchased from Gibco BRL (Carlsbad, CA, USA). The antibodies used for immunoblots are listed in Table S1.

### 2.2. Cell Culture

HEK293 cells (ATCC, USA) wild-type and stably transfected with APPsw (293sw cells) were cultured as previously described [15]. The cells were maintained in DMEM supplemented with 10% heat-inactivated FBS, 1% antibiotic-antimycotic, and 1 μg/mL G418 sulfate solution at 37 °C under a humidified 5% CO_2_ atmosphere. For immunoblot and Aβ enzyme linked immunosorbent assay (ELISA) experiments, 4 × 10^5^ cells were seeded in 35 mm dishes or a well from 6-well plate, and 3 × 10^4^ cells were seeded in a well of a 48-well plate for other ELISA experiments and MTT and lactate dehydrogenase (LDH) analyses. The cells were treated with DAPG and other chemicals at the indicated doses 8 to 24 h after seeding. The cell morphology was assessed using an Eclipse TS100 inverted microscope equipped with a DS-L3 CCD camera unit (Nikon, Japan).

### 2.3. Animals

Double transgenic APPsw/PS1dE9 mice carrying the genes encoding *APPsw* and exon-9-deleted (*dE9*) presenilin-1 (*PS1*) were purchased from the Jackson Laboratory and maintained as double hemizygotes by crossing with wild-type C57BL/6J mice, as previously reported [16]. Experiments involving animals followed the guidelines approved by the local ethical committee, the Hallym Animal Research Committee at Hallym University, and complied with the animal care guidelines of the National Institutes of Health (NIH) “Guide for the Care and Use of Laboratory Animals” (# Hallym2018-46). The animals were provided with a commercial diet and water ad libitum and were kept under controlled temperature, humidity, and lighting conditions (22 °C ± 2 °C, 55% ± 5%, and a 12/12 h light/dark cycle, respectively). Mouse genotypes were confirmed by PCR using the following primers: mouse prion protein (PrP) forward (5′-CCTCTTTGTGACTATGTGGACTGATGTCGG-3′) and PrP reverse (5′-GTGGATAACCCCTCCCCCAGCCTAGACC-3′) for internal control amplification and human APP forward (5′-GACTGACCACTCGACCAGGTTCTG-3′), and human APP reverse (5′-CTTGTAAGTTGGATTCTCATATCCG-3′) for APP transgene amplification.

### 2.4. Primary Neuron and Astrocyte Culture

Primary neurons were prepared from the cerebral cortex and hippocampus of APPsw/PS1dE9 mouse embryos at embryonic day 16, as described previously [16]. The embryos were obtained by crossing transgenic and wild-type mice. Consequently, a mixed population of wild-type and transgenic neurons was obtained, and the presence of transgenic cell populations was confirmed by PCR of the transgene from the embryonic tissues remaining after cortical and hippocampal dissection. After dissection, cortical and hippocampal tissues were dissociated by trypsinization with 0.25% trypsin-EDTA (GIBCO) and DNase I (Roche, Welwyn Garden, UK) for 10 min at 37 °C. The cells were plated on poly-D-lysine- and laminin- (Sigma) coated 6- or 48-well plates and were cultured in serum-free Neurobasal media (Invitrogen) containing B27 supplements and 1× antibiotics. The medium was replaced 4 h after cell seeding and changed every 3 days. On the 13th day of culture, neurons were treated with DAPG and other chemicals for 8 h. ELISA and immunoblot analyses were subsequently performed.

Primary astrocytes were obtained from the cerebral cortex and hippocampus of 1–3-day-old APPsw/PS1dE9 mice generated as those used to isolate primary neurons. The mixed cortical and hippocampal cells from each mouse were plated into a T75 culture flask and the genotype was determined from the remaining tissues. The transgenic cells were grown in DMEM-F12 (GIBCO) supplemented with 10% FBS. To reduce the microglial population, the flask was vigorously tapped before changing the media to remove floating microglial cells every 2 days. After 2 weeks, astrocytes were trypsinized and subcultured into 60 mm dishes or 48-well plates. Confluent astrocytes were treated with DAPG and other chemicals.

### 2.5. Intrahippocampal Stereotaxic Injection of DAPG

DAPG was injected in the hippocampus of 8-month-old APPsw/PS1dE9 mice (n = 7). The animals were anesthetized with isoflurane (2% induction, 1–1.5% for surgery and maintenance in a 65:35 mixture of N_2_O:O_2_) and secured in a stereotaxic frame (Stoelting, Wood Dale, IL, USA). Twenty nanomoles DAPG in a 2 μL solution of 15% dimethylformamide and 85% 1× DPBS or the solution alone were injected into the right or left hippocampus, respectively (2 mm posterior, 1.25 mm lateral, 2 mm depth). The injections were performed using a 33-gauge Neuros syringe (Hamilton, OH, USA) and microsyringe pump (KD Scientific, Holliston, MA, USA) at an infusion rate of 0.15 μL/min. Two days after the injection, the mice were sacrificed, and the control and DAPG-injected hippocampi were dissected and processed for immunoblot and ELISA analyses.

### 2.6. Immunoblot Analysis of Cells and Brain Tissues

After treatment, the cells were harvested and extracted in lysis buffer, i.e., 10 mM Tris buffer (pH 7.4) containing 1% sodium dodecyl sulfate (SDS) and 1 × protease inhibitor and phosphatase inhibitor cocktail (GenDEPOT, Seoul, Korea). The extracts were centrifuged at 16,000× *g* for 20 min after a brief sonication. The protein concentration in the cell lysates was determined using the bicinchoninic acid (BCA) protein assay kit. For sAPP analyses, the culture medium was used after centrifugation at 1,000× *g* for 10 min. The lysates or media were boiled in the presence of β-mercaptoethanol and SDS for 5 min and were loaded on 6–15% SDS-polyacrylamide gels. The separated proteins were transferred to polyvinylidene fluoride membranes (Millipore, MA, USA). The membranes were blocked with 5% skim milk in Tris-buffered saline (TBS, 10 mM Tris and 150 mM NaCl, pH 7.5) containing 0.05% Tween-20 for 2 h at room temperature. Afterward, they were incubated overnight with primary antibodies at 4 °C and then with the appropriate horseradish peroxidase (HRP)-conjugated secondary antibody for 1 h at room temperature. For enhanced chemoluminescence (ECL, GE Healthcare, Chalfont St Giles, UK) detection, immunoreactive bands were visualized using the LAS4000 chemiluminescence system (GE Healthcare). The image J program (NIH, Bethesda, MA, USA) was used to analyze band intensities.

Triton-soluble and insoluble fractions of brain tissues were obtained using Triton X-100 and guanidine hydrochloride (GuHCl) as detergents. First, the dissected tissues were lysed and briefly sonicated in 1 × phosphate-buffered saline (PBS), 1% Triton X-100, and 1 × protease and phosphatase inhibitor cocktail (GenDEPOT) and were kept on ice for 30 min. After centrifugation at 16,000× *g* for 30 min at 4 °C, the supernatants, designated as Triton-soluble fractions, were aliquoted and stored at −70 °C. The pellets were lysed with 6 M GuHCl for 2 h with intermittent vortexing every 30 min and were then diluted 20 times with ice cold 1× PBS. After centrifugation at 16,000× *g* for 30 min at 4 °C, the supernatant constituted the Triton-insoluble or GuHCl-soluble fraction. The Triton-soluble fraction was analyzed by immunoblotting and both Triton-soluble and -insoluble fractions used for Aβ ELISA assay.

### 2.7. Aβ ELISA Assays

To determine Aβ_1-40_ and Aβ_1-42_ levels in the cell culture medium, human Aβ sandwich ELISA kits were used following the manufacturer’s protocol (Invitrogen, Carlsbad, CA, USA). Briefly, appropriately diluted culture medium was incubated overnight at 4 °C with an antibody recognizing the terminus of Aβ opposite to that recognized by the antibody coating the 8-well strip. We used antibodies binding the N-terminal (1-x) and C-terminal (x-40 or x-42) region of Aβ. After washing, the plates were incubated with the HRP-labeled secondary antibody for 1 h at room temperature and then the tetramethylbenzidine (TMB) solution for 30 min. Finally, the stop solution was added. The optical density was measured at 450 nm using an Infinite M200 PRO microplate reader (Tecan, Zürich, Switzerland). To determine Aβ intracellular levels, the cells were lysed with TBS buffer containing 1% Triton X-100 and a protease inhibitor cocktail. The lysates were mixed with sample dilution buffer, and 20–50 μg proteins were used for the ELISA assay as described above. To analyze the Aβ content of brain tissues, we first determine the appropriate dilutions of the Triton-soluble and insoluble fractions. We analyzed a diluting range of the samples, and Aβ tissue levels were calculated using the ratio of Aβ to the total protein weight considering the dilution factor.

### 2.8. MTT Reduction and LDH Release Assays

To assess the cell viability after treatment with various compounds, MTT reduction and LDH release assays were performed as previously described [15]. For the MTT assay, cells were incubated in a 1 mg/mL MTT solution in 1× PBS in a 48-well culture plate for 1 h. Afterward, the medium was aspirated, and cells were lysed with dimethyl sulfoxide containing 0.05% NaOH. MTT values were measured using an Infinite M200 PRO microplate reader at 570 nm. An LDH cytotoxicity detection kit (Takara, Kusatsu, Japan) was used to measure LDH release. After removing cells and debris by centrifugation, culture media were mixed with the colorimetric substrates from the kit and incubated at room temperature for 0.5–1.5 h. Absorbances were measured using an Infinite M200 PRO microplate reader, and the absorbance measured at 692 nm was subtracted from the absorbance at 492 nm.

### 2.9. Aβ Clearance or Degradation Assay

To determine whether DAPG induced the degradation of exogenous and endogenous Aβ, two types of Aβ degradation assays were performed. The first type of assay was conducted after treating HEK293 cells directly with exogenous Aβ to assess the extracellular degradation and endocytosis of Aβ. In this case, 10 ng of human Aβ_1–40_ was added to the medium of each well of a 48-well plate containing HEK293 cells in the presence or absence of 50 μM DAPG. After 8 h, residual Aβ in the medium was analyzed using sandwich Aβ ELISA kit. A second type of degradation assay was conducted after incubating Aβ with or without DAPG into the culture medium collected from untreated cells to measure Aβ degradation mediated by extracellular enzymes. In this case, 1 ng of Aβ was incubated into the cell-free medium collected from controls or cells treated with 0–100 μM DAPG. After incubation for 8 h at 37 °C, the mixture of Aβ and medium was analyzed using a sandwich Aβ ELISA kit.

### 2.10. Isolation of Plasma Membrane and Other Micro-Organelles

To analyze the effects of DAPG on sAPPα intracellular localization, the plasma membrane and other micro-organelles were isolated by fractionation. Plasma membrane proteins were isolated using the Minute plasma membrane protein isolation kit (Invent, Eden Prairie, MN, USA) following the manufacturer’s instructions. Briefly, 2 × 10^6^ cells were homogenized on ice in buffer A containing a protease inhibitor cocktail for 10 min and placed in a filter cartridge. After centrifugation at 16,000× *g*, the pellets were resuspended and centrifuged at 700× *g* to remove the nuclear fraction and non-lysed cells. The supernatant was again centrifuged at 16,000× *g*. The resultant pellet constituted the fraction enriched with total membranes, whereas the supernatant was the cytosol-enriched (CE) fraction. Four-fifths of the volume of the total membrane-enriched fraction (TME) were resuspended in buffer B and centrifuged at 7800× *g* for 20 min. The pellet contained organelle membranes. The supernatant was again centrifuged at 16,000× *g* for 30 min, and the final pellet constituted the plasma membrane-enriched fraction. All CE and TME fractions were stored at −70 °C. The fractionation methods employed to isolate other micro-organelles are described in the supplementary information.

### 2.11. Analysis of In Silico Permeability of Lipid Bilayer

The lipid bilayer permeability of each compound was predicted using online prediction software ADMETlab available at http://admet.scbdd.com [17], accessed on 26 July 2022.

### 2.12. Analysis of In Silico Permeability of the BBB

The BBB permeability to DAPG was predicted using online BBB prediction software available at http://www.cbligand.org/BBB [18], accessed on 26 July 2022.

### 2.13. Statistical Analysis

The data are presented as the means ± SEM, and p-values were obtained with Student’s *t*-test using Excel 2013 software (Microsoft, Redmond, WA, USA). The differences between control and DAPG-injected hippocampi in the intrahippocampal injection experiments were compared using a paired *t*-test. For multiple comparisons, one-way analysis of the variance followed by Tukey’s test was performed using SPSS 12 software (IBM SPSS Inc., Chicago, IL, USA).

## 3. Results

### 3.1. Identification of Commercially Available Phloroglucinol Derivatives Decreasing Aβ Levels

**Figure 1:**
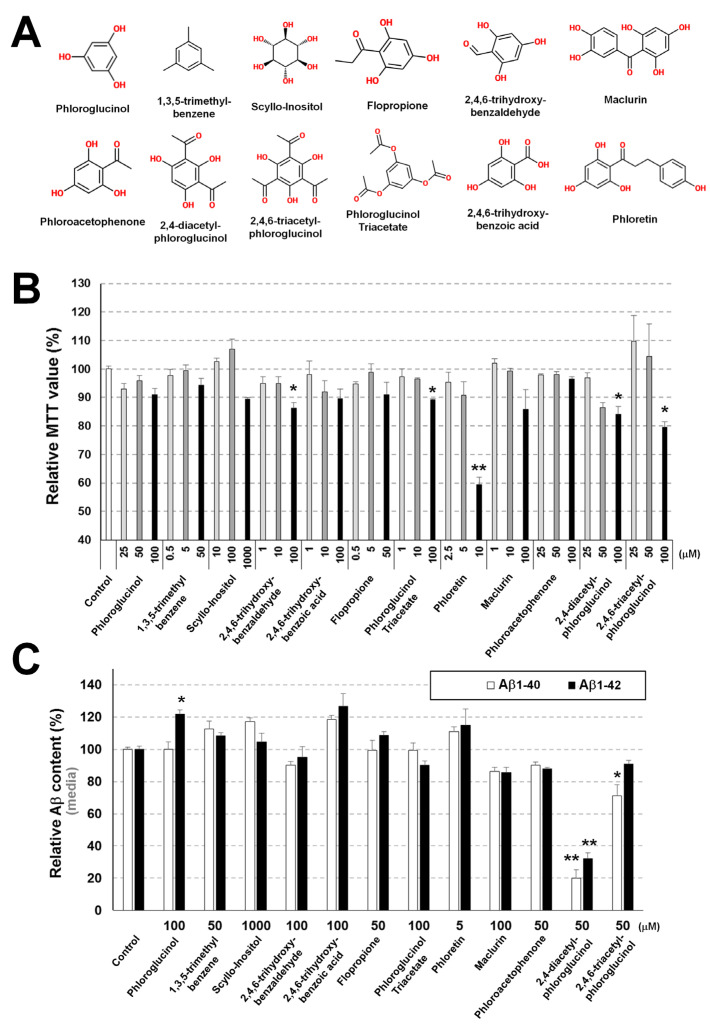
Screening for low molecular weight and commercially available phloroglucinol derivatives decreasing Aβ levels. (**A**) Chemical structures of the phloroglucinol derivatives screened in the present study. (**B**) MTT reduction assay analysis after treatment of 293sw cells with different doses of the compounds for 8 h. The column color indicates the low (light gray), medium (gray), and high (black) doses of each individual compound. The concentration range for each compound was determined through 3-4 independent repeated measurement. (**C**) ELISA analysis of Aβ_1–40_ and Aβ_1–42_ levels in the culture medium of 293sw cells treated with the indicated doses of compounds for 8 h. Values in panels B and C are presented as means ± SEMs (n = 3). Statistical significance of the differences between treated cells and untreated controls were determined using Student’s *t*-test (* *p* < 0.05, ** *p* < 0.01).

To identify commercially available phloroglucinol derivatives lowering Aβ levels, 293sw cells were incubated for 8 h with three different doses of the compounds presented in Figure 1A. The compounds, except scyllo-inositol, were predicted to exhibit optimal property for lipid bilayer permeability (Table 1). The compound toxicity was assessed using MTT reduction assays (Figure 1B). The maximal compound concentration not toxic for the cells was used to treat the cells for 8 h and determine Aβ_1–40_ and Aβ_1–42_ levels in the medium by ELISA (Figure 1C). Among all compounds, only DAPG significantly reduced Aβ_1–40_ and Aβ_1–42_ levels detected in the medium, whereas TAPG reduced only Aβ_1–40_ levels. Other phloroglucinol derivatives did not decrease Aβ amounts.

### 3.2. Comparison of the Effects on Aβ of Acetylphloroglucinol Derivatives

**Figure 2:**
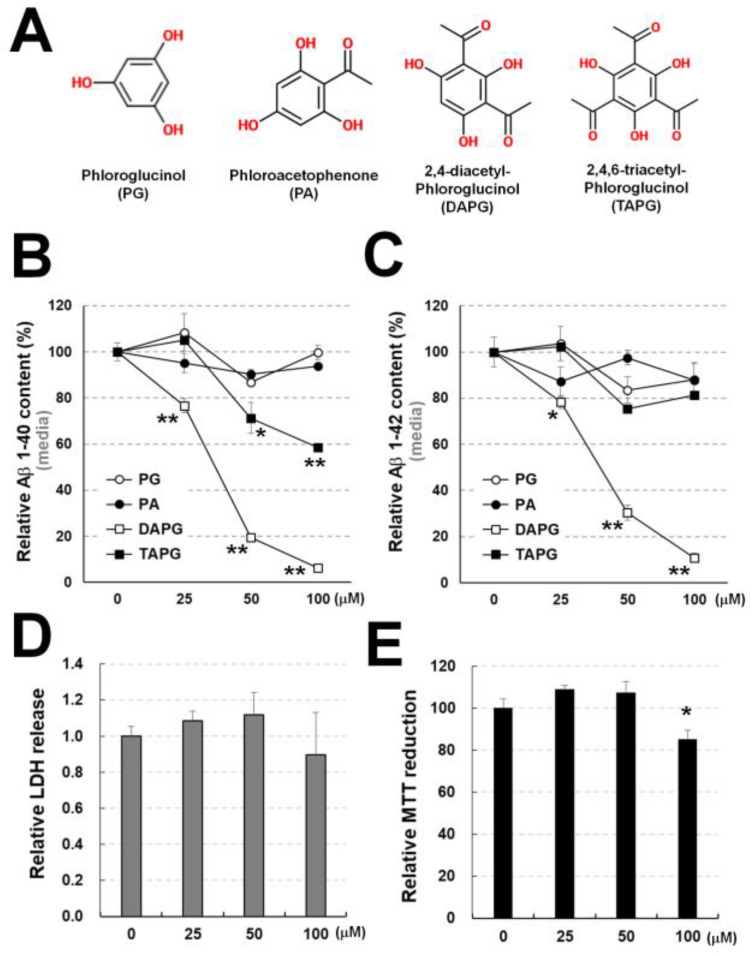
Examination of the potential of phloroglucinol and acetylphloroglucinols to lower Aβ levels. (**A**) Chemical structures of phloroglucinol and acetylphloroglucinols. (**B**,**C**) ELISA analysis of Aβ_1–40_ (**B**) and Aβ_1–42_ (**C**) levels in the culture medium of 293sw cells treated with the indicated doses of the compounds for 8 h. (**D**,**E**) LDH release (**D**) and MTT reduction (**E**) assays after treatment with the indicated doses of DAPG for 8 h. Values in panels B–E are presented as means ± SEMs (n = 3). Statistical significances of the differences between treated cells and untreated controls were determined using Student’s *t*-test (* *p* < 0.05, ** *p* < 0.01).

Since DAPG and TAPG are acetylphloroglucinols reducing secreted Aβ amounts in the medium, we compared the effects of various doses (0–100 μM) of phloroglucinol and acetylphloroglucinols (Figure 2A). Phloroglucinol and phloroacetophenone did not affect Aβ_1–40_ and Aβ_1–42_ levels measured by ELISA (Figure 2B,C). TAPG concentrations of 50 μM or higher decreased Aβ_1–40_, but not Aβ_1–42_, levels. All tested DAPG concentrations reduced Aβ_1–40_ and Aβ_1–42_ amounts secreted into the medium without affecting LDH release (Figure 2D). MTT reduction was decreased by 100 μM DAPG (Figure 2E), indicating a growth arrest rather than cell death.

### 3.3. DAPG Reduces Aβ Production and Increases sAPPα Production and Secretion in 293sw Cells

**Figure 3:**
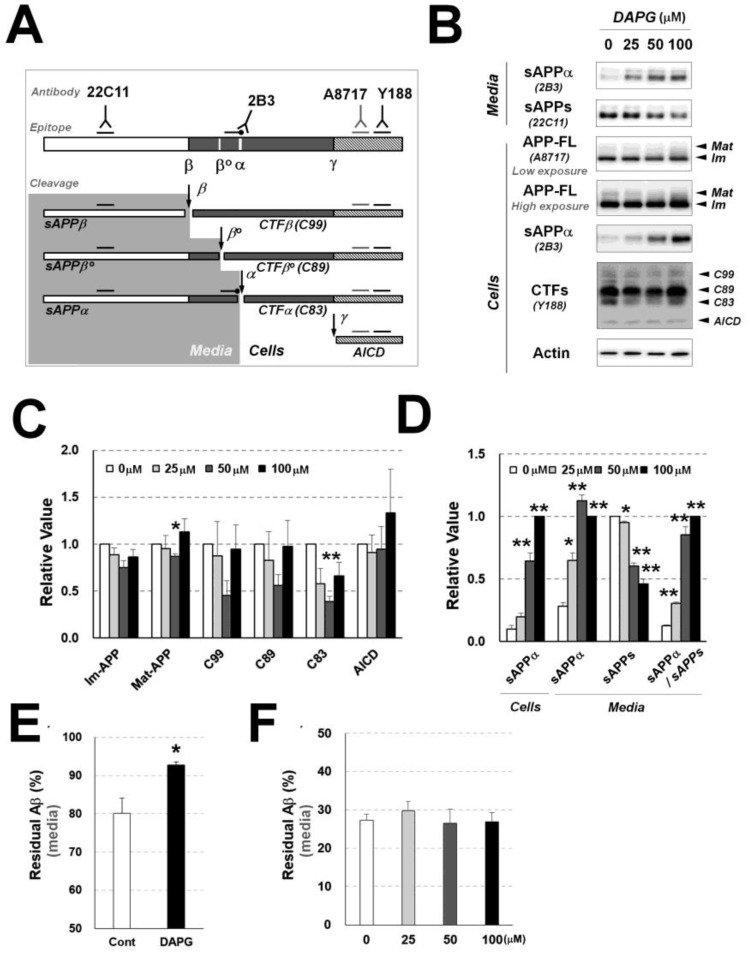
Examination of DAPG-mediated regulation of Aβ production and degradation or clearance in 293sw cells. (**A**) Schematic illustration of APP processing and antibodies detecting the cleaved products. Full-length APP (APP-FL) can be cleaved into soluble APPα (sAPPα) and CTFα (C83) by α-secretase, soluble APPβ (APPβ) and CTFβ (C99) by β-secretase, or soluble APPβ’ (APPβ’) and CTFβ’ (C89) by β-secretase. C99 and C89 can be cleaved into APP intracellular domain (AICD) and Aβ by γ-secretase and C83 cleavage by γ-secretase produces AICD and non-toxic p3 fragment. Alternatively, non-cleaved APP-FL can be glycosylated, and the glycosylated form of APP-FL is mature form (Mat-APP) and non-glycosylated form is immature form (Im-APP). To examine APP processing by immunoblot, 22C11 and α-cleavage site specific 2B3 antibody detect sAPPs (sAPPα, sAPPβ’, and sAPPβ) and sAPPα in culture medium, respectively. In cell lysates, A8717 detects APP-FL (Mat- and Im-APP), 2B3 detects intracellular sAPPα, and Y188 detects CTFs including AICD. (**B**) Immunoblot analysis of APP processing in 293sw cells and culture medium after 8 h treatment with 0–100 μM DAPG. The levels of sAPPα and sAPPs in the medium and APP-FL (Mat- and Im-APP), sAPPα, CTFs (C99, C89, and C83), AICD, and actin in cells were examined. (**C**,**D**) Densitometry analysis of the immunoblot bands for the markers described in (**B**) in 293sw cells (**C**) and for sAPPα and sAPPs in the cells and culture medium (**D**). (**E**) Exogenous Aβ clearance assay on HEK293 cells. ELISA analyses of the culture medium were performed after treating wild-type HEK293 cells with 10 ng Aβ_1–40_ with or without DAPG for 8 h. (**F**) Exogenous Aβ degradation analysis of the culture medium from untreated and DAPG-treated HEK293 cells. Cell-free medium collected from HEK293 cells treated with 0–100 μM DAPG for 8 h was incubated with 1 ng Aβ_1-40_ at 37 °C for 8 h. Values in panels C–F are presented as means ± SEMs (n = 3). Statistical significances of the differences between treated groups and untreated controls were determined using Student’s *t*-test (* *p* < 0.05, ** *p* < 0.01).

To determine the mechanisms involved in the DAPG-mediated reduction in Aβ levels, we analyzed DAPG effects on APP processing and Aβ production (Figure 3A–D). The levels of total APP and its intracellular fragments were not significantly changed by DAPG except those of the mature form of APP (Mat-APP) and C83, a C-terminal fragment generated by α-secretase (CTFα), which were affected by 50 μM DAPG (Figure 3B,C). Regarding APP fragments secreted into the medium, the levels of soluble APPα (sAPPα), which is an extracellular fragment generated by α-secretase, were increased by the cell treatment with 25–100 μM DAPG, whereas total soluble APP (sAPPα + sAPPβ) levels were decreased by DAPG, and the ratio of sAPPα to sAPPs was increased (Figure 3D). The amounts of intracellular and secreted sAPPα were also increased by DAPG. These data suggested that intracellular sAPPα targeted by DAPG was located in intracellular microorganelles or vesicles such as the endosome and secretory vesicles involved in endocytosis and exocytosis.

To determine whether DAPG reduced Aβ levels by activating Aβ degradation or clearance [1,4], two types of experiments were conducted. First, to measure directly cellular Aβ clearance and degradation, exogenous Aβ was co-incubated with DAPG-treated and untreated wild-type HEK293 cells, which produce low Aβ amounts compared with those in 293sw cells (Figure 3E). Next, to measure the degradation of Aβ by secretable extracellular Aβ degradation enzymes such as matric metallopeptidase 9 (MMP-9) and cathepsin B [19,20], exogenous Aβ was mixed and incubated with medium collected from control and DAPG-treated wild-type HEK293 cells (Figure 3F). The levels of exogenous Aβ after incubation with cells (Figure 3E) or the medium collected from control and DAPG-treated cells (Figure 3F) were not reduced by DAPG treatment. Considering these data and DAPG effects on α-secretase-mediated APP processing and sAPPα secretion, DAPG likely reduced Aβ production but not its degradation or clearance.

### 3.4. DAPG-Mediated sAPPα Increase Occurs in Intracellular Organelles, except the Endosome, and Not in the Plasma Membrane of 293sw Cells

**Figure 4:**
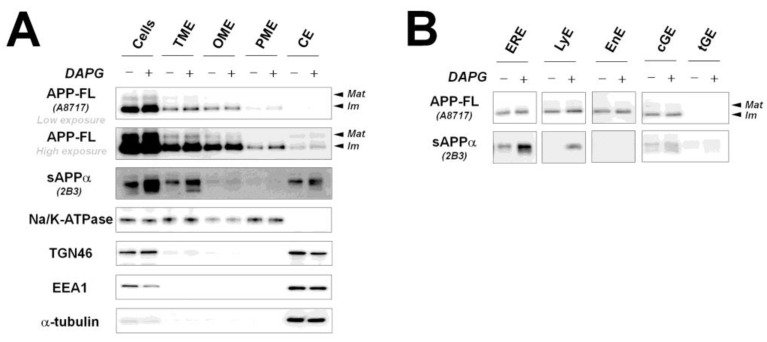
Examination of APP-FL and sAPPα levels in intracellular organelles after DAPG treatment. (**A**) Changes of APP-FL and sAPPα levels induced by DAPG in total cell lysate (Cells, 8 μg), total membrane-enriched (TME, 4 μg), organelle membrane-enriched (OME, 2 μg), plasma membrane-enriched (PME, 2 μg), and cytosol-enriched (CE, 6 μg) fractions. Markers of intracellular organelles including the plasma membrane (Na/K-ATPase), trans-Golgi (TGN46), early endosome (EEA1), and cytosol (α-tubulin) were analyzed. (**B**) Changes of APP-FL and sAPPα levels induced by DAPG in the ER-enriched (ERE, 1 μg), lysosome-enriched (LyE, 1 μg), cis-Golgi-enriched (cGE, 1 μg), and trans-Golgi-enriched (tGE, 1 μg) fractions.

It has been reported that α-secretase is mainly located in the plasma membrane and β- and γ-secretases are activated in intracellular organelles such as the endosome and Golgi complex [21,22]. Because our data revealed that DAPG increased intracellular and extracellular sAPPα levels, we examined the cellular localization of sAPPα regulated by DAPG by isolating intracellular organelle- and plasma membrane-enriched fractions (Figure 4A). DAPG did not significantly change the levels of full-length APP (APP-FL) in TME, organelle membrane-enriched (OME), and CE fractions compared with those measured in untreated controls. In contrast, sAPPα levels were increased by DAPG in TME, OME, and CE fractions, indicating that intracellular sAPPα production or accumulation occurred in the lumen of intracellular organelles or vesicular structures that are connected with the extracellular space rather than in the cytosol. We detected sAPPα in the CE fraction, although this fraction does not usually contain sAPPα. This was likely due to the presence in the CE fraction of small vesicles including structures positive for TGN46 and EEA1, which are markers of the trans-Golgi network (TGN) and early endosome, respectively, in which sAPPα levels were strongly increased by DAPG. Therefore, we further examined sAPPα levels in isolated intracellular organelles (Figure 4B).

To this aim, ER-enriched (ERE), lysosome-enriched (LyE), endosome-enriched (EnE), cis-Golgi complex-enriched (cGE), and trans-Golgi complex and secretory vesicle-enriched (tGE) fractions were isolated. DAPG did not appear to change APP-FL levels in any fractions. However, sAPPα levels were increased in all fractions except EnE fraction. Therefore, DAPG increased sAPPα levels likely by acting on the intracellular machinery, but not the plasma membrane, and the intracellular sAPPα production or accumulation occurred in ER, lysosomes, and Golgi complex, but not in endosomes. This indicated that intracellular trafficking might be a major target of DAPG to modify sAPPα and Aβ levels.

### 3.5. DAPG Reduces Aβ Production and Increases α-Secretase-Mediated APP Processing and sAPPα Secretion in APPsw/PS1dE9-Derived Primary Astrocytes

**Figure 5:**
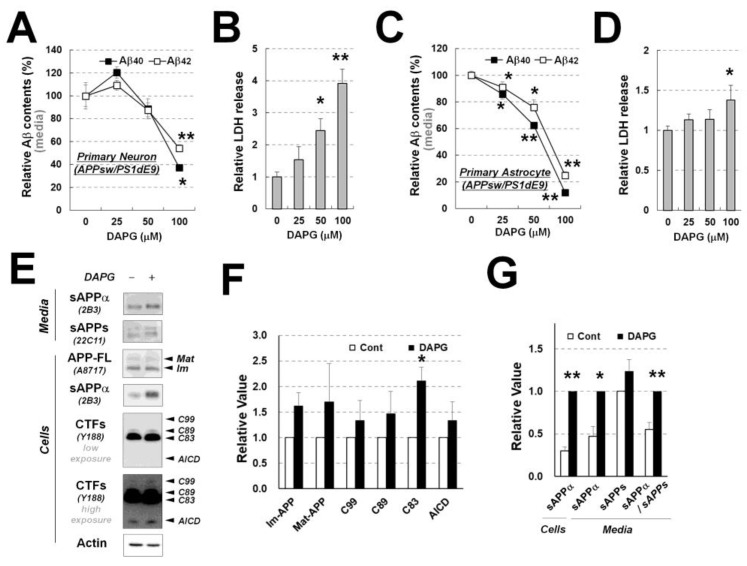
Examination of DAPG-mediated decrease in Aβ levels and toxicity in primary neurons and astrocytes from APPsw/PS1dE9 transgenic mice. (**A**,**B**) ELISA analysis of Aβ_1–40_ and Aβ_1–42_ levels (**A**) and LDH release analysis (**B**) in the culture medium from primary neurons isolated from APPsw/PS1dE9 transgenic mice. Neurons were treated with 0–100 μM DAPG for 8 h. (**C**) ELISA analysis of Aβ_1–40_ and Aβ_1–42_ levels (**C**) and LDH release analysis (**D**) in the culture medium from primary astrocytes isolated from the transgenic mice. Astrocytes were treated with 0–100 μM DAPG for 8 h. (**E**) Immunoblots analysis of APP processing in primary astrocytes and culture medium after 8 h treatment with 30 μM DAPG. The levels of sAPPα and sAPPs in the medium and immature (Im) and mature (Mat) APP-FL, sAPPα, CTFs, AICD, and actin in cells were examined. (**F**,**G**) Densitometry analysis of markers in primary astrocytes (**F**) and sAPPs in the cells and medium (**G**) from immunoblots presented in (**E**). Values in all graphs are presented as means ± SEMs (n = 3). Statistical significances of the differences between treated groups and untreated controls were determined using Student’s *t*-test (* *p* < 0.05, ** *p* < 0.01).

To determine whether DAPG also decreased Aβ levels in primary neurons or astrocytes derived from an AD animal model, primary neurons and astrocytes were isolated from APPsw/PS1dE9 transgenic mice. These cells were treated with DAPG, as were 293sw cells. In transgenic primary neurons, 100 μM DAPG reduced Aβ secretion (Figure 5A). However, significantly more (2–4 folds) cell death was measured by LDH release for DAPG concentrations of 50–100 μM compared with that in untreated controls (Figure 5B). In contrast, DAPG significantly reduced secreted Aβ levels for concentrations of 25–100 μM in transgenic primary astrocytes (Figure 5C), and LDH release was only slightly increased by 100 μM DAPG (Figure 5D). Next, we examined whether APP processing was affected by DAPG in primary astrocytes. C83 levels were increased by 30 μM DAPG (Figure 5E,F), and intracellular and secreted sAPPα amounts were also greater after DAPG treatment, whereas sAPPs secretion was not significantly changed (Figure 5E,G). Thus, there were significant differences in DAPG effects in transgenic primary astrocytes compared with those observed in 293sw cells (Figure 3), particularly regarding the increased C83 levels and unchanged sAPPs secretion.

### 3.6. Intrahippocampal Injection of DAPG Reduces Triton-Soluble Aβ and Increases sAPPα Levels in APPsw/PS1dE9 Transgenic Animal Models

To determine the in vivo effects of DAPG, 20 mg/kg DAPG were injected intraperitoneally into APPsw/PS1dE9 transgenic mice daily for 9 days, and a Morris water maze test was performed (Figure S1A). DAPG treatment had no impact on the spatial memory decline of the transgenic mice. DAPG also did not affect APP processing, including APP-FL, sAPPα, CTFs formation, in the hippocampus of transgenic mice (Figure S1B,C). In addition, computational simulation of DAPG penetrance of the BBB showed that DAPG could not cross the BBB (Figure 1A), indicating that ineffectiveness of systemic treatment of DAPG may be due to BBB impermeability.

**Figure 6:**
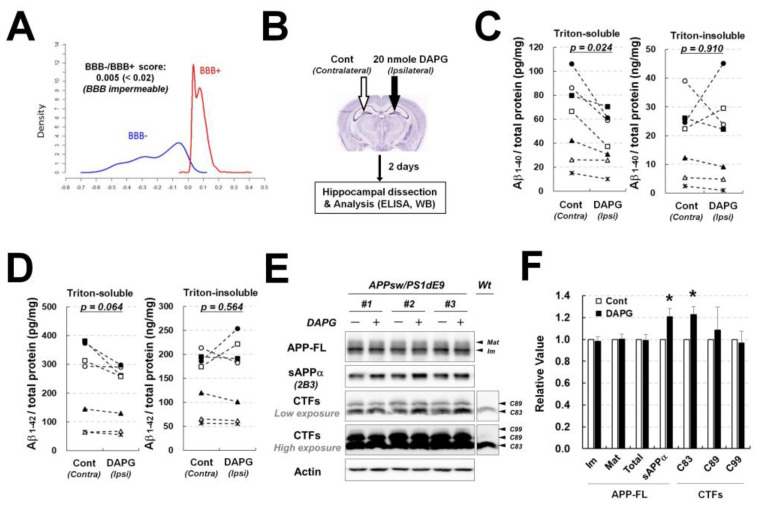
Examination of DAPG effects on Aβ levels and APP processing after DAPG intrahippocampal injection. (**A**) Computational prediction of blood–brain barrier permeability to DAPG using online prediction software available at http://www.cbligand.org/BBB, accessed on 26 July 2022. (**B**) Schematic illustration of DAPG intrahippocampal injection and further analyses, including immunoblot and Aβ ELISA assays, in 8-month-old APPsw/PS1dE9 transgenic mice. Detailed methods for DAPG injection and Aβ analysis are described in Materials and Methods. (**C**,**D**) ELISA analysis of Aβ_1–40_ (**C**) and Aβ_1–42_ (**D**) levels obtained in Triton-soluble and Triton-insoluble (or GuHCl-soluble) fractions from control and DAPG-injected hippocampi. In the graph, the values obtained for control (contralateral) and DAPG-injected (ipsilateral) hippocampi were statistically paired. Paired *t*-tests were used to determine the statistical significance of the differences (n = 7) and the resultant p-values are plotted on the graph. (**D**,**E**) Immunoblot analysis of APP processing (**E**) in control and DAPG-injected hippocampi from the transgenic mice and densitometry analysis of the immunoblots (**F**). Values in (**F**) are presented as means ± SEMs (n = 7). Statistical significances of difference between the injected and uninjected groups were determined using paired *t*-test (* *p* < 0.05).

As an alternative to investigated DAPG in vivo effects, DAPG was injected directly into the hippocampus of transgenic mice (Figure 6B) and Aβ levels in Triton-soluble and Triton-insoluble (or GuHCl-soluble) fractions from the contralateral control and ipsilateral DAPG-injected hippocampi were analyzed to determine the amount of soluble (or initially secreted Aβ) and insoluble (or accumulating extracellular Aβ that aggregated into amyloid plaques) Aβ, respectively (Figure 6C,D). Compared with the amounts found in control hippocampi, Triton-soluble Aβ_1–40_ levels were significantly reduced, and Aβ_1–42_ amounts were slightly, albeit not significantly (*p* = 0.064), decreased by DAPG injection in the hippocampus. However, DAPG injection did not affect Aβ content of the Triton-insoluble fraction, suggesting that DAPG affected early events of Aβ accumulation in tissues such as Aβ secretion or production rather than late processes such as aggregation and plaque formation. Immunoblot analyses of untreated and DAPG-injected hippocampi revealed that sAPPα and C83 levels were significantly increased by DAPG similarly to what was observed in primary astrocytes treated with DAPG (Figure 5 and Figure 6E,F).

### 3.7. ADAM10 Inhibition Blocks DAPG Effects on sAPPα Production but Not on Secreted Aβ Levels in 293sw Cells and Transgenic Primary Astrocytes

**Figure 7:**
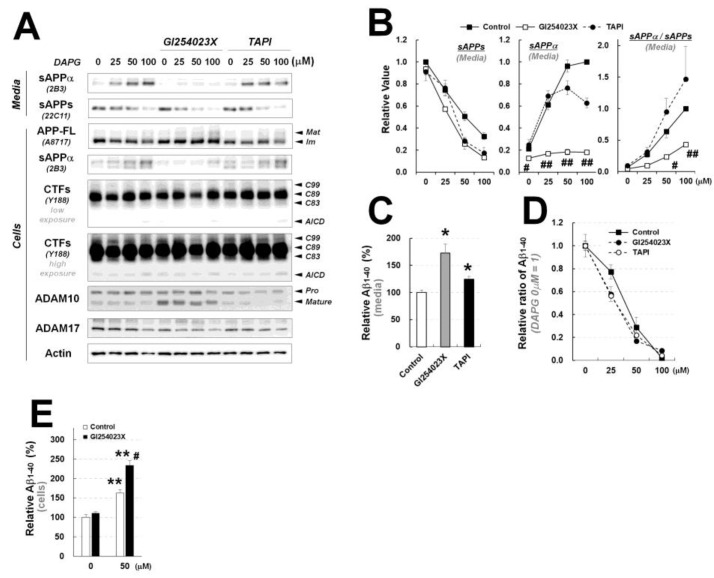
Examination of the effects of ADAM10 and ADAM17 inhibitors on DAPG-mediated decrease in Aβ levels and changes in APP processing in 293sw cells. (**A**) Immunoblot analysis of APP processing and expression of ADAM10 and ADAM17 in 293sw cells co-treated with 0–100 μM DAPG and 10 μM GI243023X (an ADAM10 inhibitor) or 10 μM TAPI (an ADAM17 inhibitor) for 8 h. (**B**) Densitometry analyses of the immunoblots from (**A**) for sAPPα and sAPPs levels in the culture medium and the ratio of sAPPα to sAPPs levels. Values are presented as means ± SEMs (n = 3). Statistical significances of the differences between DAPG-treated group and both DAPG- and GI254023X-treated group (^#^ *p* < 0.05, ^##^ *p* < 0.01) were determined using one-way ANOVA followed by Tukey’s test. (**C**) ELISA analysis of Aβ_1–40_ levels in the medium of 293sw cells treated with GI254023X or TAPI for 8 h. Values are presented as means ± SEMs (n = 3). Statistical significances of the differences between treated groups and untreated controls were determined using Student’s *t*-test (* *p* < 0.05). (**D**) Relative ratio of Aβ_1–40_ in the medium of 293sw cells compared with DAPG-untreated group determined by ELISA analysis after treatment with 0–100 μM DAPG with or without GI254023X and TAPI for 8 h. (**E**) ELISA analysis of intracellular Aβ_1–40_ levels after treatment of 293sw cells with 50 μM DAPG in presence or absence of GI254023X for 8 h. Values are presented as means ± SEMs (n = 3). Statistical significances of the differences between DAPG-untreated group and DAPG-treated group (** *p* < 0.01) or between DAPG-treated group and both DAPG- and GI254023X-treated group (^#^ *p* < 0.05) were determined using one-way ANOVA followed by Tukey’s test.

To further evaluate the mechanisms involved in DAPG effects on sAPPα production and Aβ levels, 293sw cells were cotreated with DAPG and GI254023X or TAPI, an ADAM10 or ADAM17 inhibitor, respectively, because ADAM10 and ADAM17 have been previously identified in vivo as α-secretase candidates [23]. Immunoblot analyses revealed that GI254023X inhibited the accumulation of intracellular and secreted sAPPα induced by DAPG, without affecting further APP processing (Figure 7A,B). TAPI co-treatment did not lead to significant changes compared with sAPPα levels present in cells treated with DAPG alone. Additionally, cell treatment with β- or γ-secretase inhibitors did not impact the effect of DAPG on sAPPα secretion (Figure S2).

Regarding Aβ levels, GI254023X and TAPI increased the basal levels of secreted Aβ (Figure 7C) but did not alter the dose-dependent reduction ratio of Aβ induced by DAPG (Figure 7D). Thus, we examined whether intracellular Aβ levels were affected by DAPG treatment alone or in combination with GI254023X (Figure 7E). Interestingly, DAPG treatment significantly increased intracellular Aβ levels, suggesting that DAPG-mediated reduction in the amounts of secreted Aβ was at least partly due to the suppression of Aβ secretion. GI254023X and DAPG co-treatment increased intracellular Aβ levels compared with those observed with DAPG. Therefore, the data suggested that ADAM10 inhibitor prevented DAPG-mediated reduction in Aβ intracellular production but not secretion.

**Figure 8:**
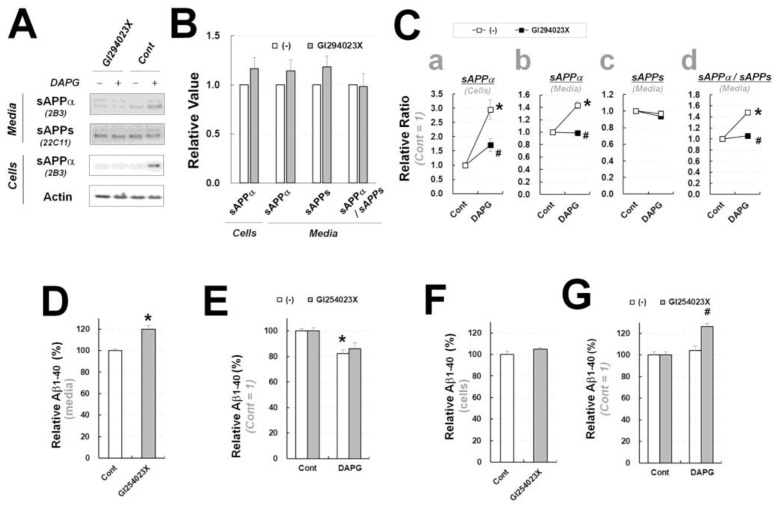
Examination of ADAM10 inhibitor effects on DAPG-mediated decrease in Aβ and increase in sAPPα levels in primary astrocytes from APPsw/PS1dE9 transgenic mice. (**A**) Immunoblot analysis of sAPPα and sAPPs levels in primary astrocytes treated with 10 μM GI243023X (ADAM10 inhibitor) and 30 μM DAPG for 8 h. (**B**,**C**) Densitometry analysis of sAPPα and sAPPs levels in the medium or cells from the immunoblots presented in (**A**). Panel B shows the effects of GI294023X alone. Panel C shows the relative ratio between controls and cells treated with DAPG or DAPG and GI294023X. The values of sAPPα levels in cells (**a**) and the medium (**b**), sAPPs levels in the medium (**c**), and the ratio of sAPPα to sAPPs levels in the medium (**d**) are represented. (**D**) ELISA analysis of Aβ_1–40_ amounts in the culture medium from primary astrocytes treated with GI294023X for 8 h. (**E**) Relative Aβ_1–40_ content in the culture medium from primary astrocytes treated with 30 μM DAPG with or without 10 μM GI294023X for 8 h measured by ELISA (**F**,**G**) ELISA analysis of intracellular Aβ_1–40_ levels in primary astrocytes treated with GI294023X alone (**F**) or GI294023X with DAPG (**G**). Statistical significances of differences between treated groups and untreated controls were determined using Student’s *t*-test in panels D and E (* *p* < 0.05). In panels C and G, differences between untreated and DAPG-treated groups (* *p* < 0.05) or between DAPG-treated and both DAPG- and GI254023X-treated group (^#^ *p* < 0.05) were analyzed using one-way ANOVA followed by Tukey’s test.

Next, we tested the effects of GI294023X on DAPG-treated primary astrocytes to determine whether ADAM10 regulates DAPG effects on sAPPα and Aβ levels in primary astrocytes isolated from transgenic mice (Figure 8). The basal levels of intracellular sAPPα and secreted sAPPα and sAPPs in primary astrocytes were not changed by GI294023X (Figure 8A,B). In contrast, ADAM10 inhibition prevented DAPG-mediated increase in intracellular and secreted sAPPα levels (Figure 8C), similarly to its effects in 293sw cells (Figure 7B). Additionally, ADAM10 inhibition increased secreted Aβ levels and did not significantly affect DAPG-mediated reduction in secreted Aβ levels in astrocytes (Figure 8D,E), which was similar to ADAM10 inhibition effects observed in 293sw cells (Figure 7C,D). Finally, intracellular Aβ levels were not changed by GI293023X, whereas GI254023X and DAPG co-treatment resulted in higher intracellular Aβ levels compared with those in DAPG-treated astrocytes (Figure 8F,G), which agreed with the data obtained in 293sw cells (Figure 7E). Finally, unlike its effects in 293sw cells (Figure 7E), treatment with DAPG alone did not change the basal intracellular Aβ levels in astrocytes (Figure 8G). Therefore, ADAM10 inhibition had similar effects on sAPPα and Aβ levels in DAPG-treated primary astrocytes and 293sw cells, but the impact of ADAM10 inhibition in absence of DAPG was different between both cell types, indicating that the basal regulation of sAPPα and Aβ might be cell-type-dependent.

To determine how the DAPG-mediated increase in sAPPα levels was affected by ADAM10, α-secretase activity was examined after DAPG treatment in 293sw cells or their membrane fraction. DAPG treatment did not modify α-secretase activity in the membrane fraction (Figure S3A) or the cultivated cells (Figure S3B). Because ADAM10 total level and maturation in 293sw cells were also not affected by DAPG (Figure 7A), DAPG might not be a direct regulator of ADAM10, and DAPG-mediated effects on sAPPα production are likely caused by indirect mechanisms such as changes of co-localization or translocation between ADAM10 and APP-FL.

### 3.8. BFA, an Intracellular Trafficking Inhibitor, Prevented DAPG-Mediated Reduction in sAPPs and Secreted AΒ Levels in 293sw Cells

**Figure 9:**
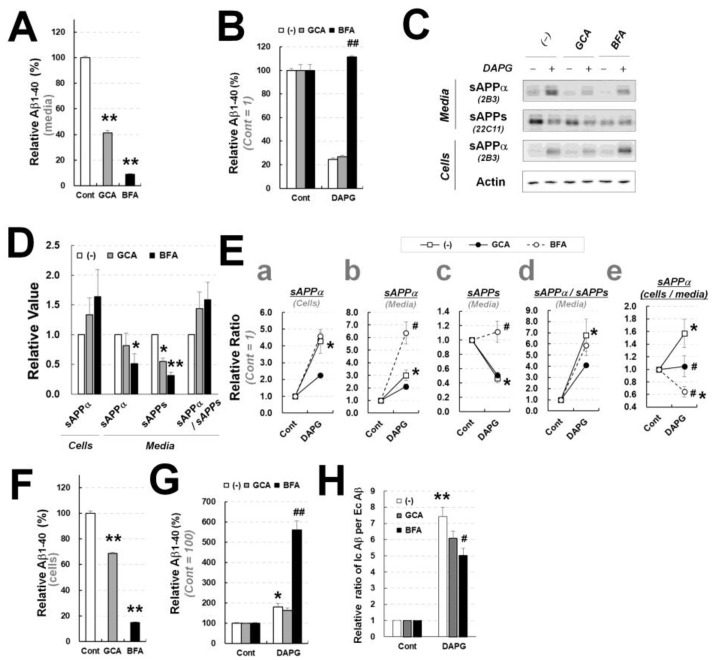
Examination of GCA and BFA effects in 293sw cells on DAPG-mediated Aβ levels decrease and sAPPα levels increase. (**A**) ELISA analysis of Aβ_1–40_ amounts in the culture medium of 293sw cells treated with 3.5 μM GCA or 60 ng/mL BFA for 8 h. (**B**) Relative Aβ_1–40_ content in the culture medium of 293sw cells treated with 50 μM DAPG with or without 3.5 μM GCA or 60 ng/mL BFA for 8 h analyzed by ELISA. (**C**) Immunoblot analysis of sAPPα and sAPPs levels in 293sw cells treated with DAPG and GCA or BFA for 8 h. (**D**,**E**) Densitometry analysis of sAPPα and sAPPs levels in the medium or cells from immunoblots presented in (**C**). Panel D represents the effects of GCA or BFA alone. Panel E shows the relative ratio between controls and cells treated with DAPG alone (untreated) or with DAPG and GCA or BFA. The values of sAPPα levels in cells (**a**) and the medium (**b**) and sAPPs levels in the medium (**c**) as well as the ratios of sAPPα to sAPPs levels in the medium (**d**) and of sAPPα levels in cells to the levels in the medium (**e**) are presented. (**F**,**G**) ELISA analysis of intracellular Aβ_1–40_ levels in 293sw cells treated with GCA or BFA (**F**) or DAPG and GCA or BFA (**G**). (**H**) Ratios of Aβ_1–40_ levels in cells treated with DAPG and GCA or BFA (Ic Aβ) to the levels in their culture medium (Ec Aβ). All values in graphs are presented as means ± SEMs (n = 3). Statistical significances of differences between untreated and DAPG-treated groups (* *p* < 0.05, ** *p* < 0.01) or between DAPG–treated and DAPG- and GCA- or BFA-treated groups (^#^ *p* < 0.05, ^##^ *p* < 0.01) were analyzed. In panels (**A**,**D**,**F**), Student’s *t*-test was used (* *p* < 0.05, ** *p* < 0.01), whereas one-way ANOVA followed by Tukey’s test was performed in panels (**B**,**E**,**G**,**H**).

To evaluate whether intracellular trafficking was involved in the DAPG-mediated modulation of Aβ and sAPPα levels, cells were treated with various inhibitors of intracellular trafficking with or without DAPG. The inhibitors targeted the ER to Golgi trafficking (GCA and BFA), intra-Golgi trafficking (ZCL278 and CI-976; inhibitors for Cdc42 and lysophospholipid transferases, respectively), lysosomes (vacuolin-1; an inhibitor for lysosomal exocytosis), anterograde vesicles (EHNA; an inhibitor for anterograde transport), and the retrograde transport from endosome to Golgi (Retro-2). Treatment of GCA, BFA, ZCL-278, or vacuolin-1 alone reduced basal secreted Aβ levels in 293sw cells (Figure 9A and Figure S4A,C). Next, compared with the DAPG-alone treatment, only the co-treatment of BFA with DAPG prevented a DAPG-mediated reduction in Aβ levels, whereas the other inhibitors with DAPG had no impact (Figure 9B and Figure S4B,D).

The effects of GCA or BFA on intracellular and secreted levels of sAPPα were examined by immunoblot analyses of sAPPα and sAPPs in 293sw cells and their culture medium (Figure 9C–E). GCA or BFA did not affect DAPG-mediated increase in intracellular sAPPα levels (Figure 9E(a)), but sAPPα and sAPPs levels in the medium of cells co-treated with DAPG and BFA were increased compared with those in the medium of DAPG-treated cells (Figure 9E(b,c)). Additionally, the ratio of sAPPα to sAPPs was not different between the medium of DAPG and BFA-cotreated and DAPG-treated cells (Figure 9E(d)), indicating that the mechanisms triggered by BFA to modulate DAPG effects on secreted Aβ levels were not associated with ADAM10. On the other hand, the ratio of intracellular sAPPα to secreted sAPPα (in the medium) revealed that DAPG significantly reduced sAPPα secretion, GCA or BFA co-treatment inhibited this reduction, BFA and DAPG co-treatment especially induced a significant decrease in sAPPα secretion compared with that observed after treatment with BFA alone (Figure 9E(e)). Thus, the BFA modulation of sAPPα levels in DAPG-treated 293sw cells might be linked to sAPPα and sAPPs (or sAPPβ) production or secretion rather than ADAM10-mediated sAPPα production. In fact, ZCL-278 treatment significantly reduced DAPG-mediated induction of intracellular and secreted sAPPα levels, which mimicked the effects of ADAM10 inhibition by GI294023X, but did not change the DAPG-mediated reduction in secreted Aβ levels (Figure S4B,E,F), suggesting independent mechanisms of action for DAPG on sAPPα and Aβ secretion in 293sw cells.

To investigate the possible associations among DAPG, BFA, and Aβ in 293sw cells, the effects of BFA treatment on intracellular Aβ levels and the ratio of intracellular to secreted Aβ were examined. GCA or BFA reduced intracellular Aβ levels (Figure 9F), similarly to their effect on secreted Aβ (Figure 9A). Co-treatment with BFA and DAPG induced a significantly greater increase in intracellular Aβ levels than that observed with DAPG alone (Figure 9G). In particular, the ratio of Aβ in cells to Aβ in medium was significantly reduced, compared with that obtained after DAPG treatment, after BFA and DAPG co-treatment, but not after GCA and DAPG co-treatment (Figure 9H). Thus, BFA, but not GCA, inhibited DAPG-mediated reduction in extracellularly secreted Aβ and rather increased DAPG-mediated intracellular Aβ accumulation. Additionally, intracellular and extracellular Aβ trafficking β by DAPG was also inhibited by BFA.

### 3.9. BFA and GCA Differentially Inhibit DAPG-Mediated Aβ Decrease and sAPPα Increase in Primary Astrocytes from Transgenic Mice

**Figure 10:**
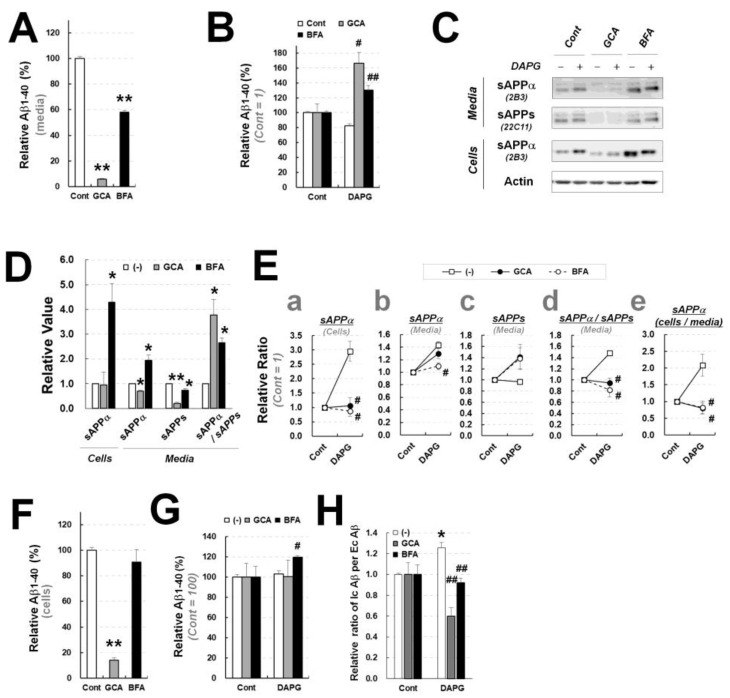
Examination of GCA and BFA effects on DAPG-mediated Aβ level decrease and sAPPα level increase in primary astrocytes from APPsw/PS1dE9 transgenic mice. (**A**) ELISA analysis of Aβ_1–40_ levels in the culture medium of primary astrocytes treated with 3.5 μM GCA or 60 ng/mL BFA for 8 h. (**B**) Relative Aβ_1–40_ content in the medium of primary astrocytes treated with 30 μM DAPG in the presence or absence of 3.5 μM GCA or 60 ng/mL BFA for 8 h obtained by ELISA analysis. (**C**) Immunoblot analysis of sAPPα and sAPPs levels in the primary astrocytes treated with DAPG and GCA or BFA for 8 h. (**D**,**E**) Densitometry analysis of sAPPα and sAPPs levels in the medium or cells from immunoblots presented in (**C**). Panel D shows the effects of GCA or BFA alone (**D**). Panel E shows the ratio between controls and cells treated with DAPG (untreated) or DAPG and GCA or BFA (**E**). The levels of sAPPα in cells (**a**) and the medium (**b**) and sAPPs in the medium (**c**) as well as the ratios of sAPPα to sAPPs levels in the medium (**d**), and of sAPPα levels in cells to those in the medium (**e**) are shown. (**F**,**G**) ELISA analysis of intracellular Aβ_1–40_ levels in primary astrocytes treated with GCA or BFA (**F**) and of relative Aβ_1–40_ content after DAPG and GCA or BFA co-treatment (**G**). (**H**) Ratios of Aβ_1–40_ levels in cells (Ic Aβ) to those in the medium (Ec Aβ) after DAPG and GCA or BFA co-treatment. Statistical significances of differences between treated and untreated groups in panels A, D, and F were determined using Student’s *t*-test (* *p* < 0.05, ** *p* < 0.01). In panels B, E, G, and H, statistical significances of differences between untreated and DAPG-treated groups (* *p* < 0.05) and between DAPG-treated and DAPG- and GCA- or BFA-treated groups (^#^ *p* < 0.05, ^##^ *p* < 0.01) were determined using one-way ANOVA followed by Tukey’s test.

To identify whether DAPG-mediated modulation of Aβ and sAPPα also involved the intracellular trafficking pathway in transgenic primary astrocytes, primary astrocytes isolated from APPsw/PS1dE9 transgenic mice were co-treated with DAPG and GCA or BFA. Basal Aβ levels in the culture medium were significantly decreased by GCA or BFA treatment (Figure 10A). Co-treatment with DAPG and GCA or BFA significantly reversed DAPG-mediated reduction in Aβ levels in the medium (Figure 10A,B). Immunoblot analyses of sAPPα and sAPPs in transgenic primary astrocytes and their medium (Figure 10C–F) revealed that co-treatment with DAPG and GCA or BFA reduced sAPPα levels in cells and medium, with the exception of sAPPα in the medium of DAPG and GCA-treated cells, and the ratio of secreted sAPPα to sAPPs (Figure 9E(a–d)), which resembled the effects of ADAM10 inhibition (Figure 8C). DAPG and GCA or BFA co-treatment also prevented the increase in the ratio of sAPPα levels in cells to sAPPα levels in medium (Figure 10E(e)). These effects of GCA and BFA on secreted Aβ and sAPPα production and secretion in DAPG-treated astrocytes were different from those observed in 293sw cells (Figure 9).

The analysis of Aβ changes induced by treatment of primary astrocytes with GCA or BFA revealed that GCA, but not BFA, reduced intracellular Aβ levels (Figure 9F). BFA and DAPG co-treatment induced significantly greater intracellular Aβ levels than those measured after treatment with DAPG alone (Figure 10G). Finally, the ratio of Aβ levels in cells to Aβ levels in medium was significantly reduced after co-treatment with DAPG and BFA or GCA compared with that after DAPG treatment (Figure 10H). Thus, the effects of GCA and BFA on DAPG-mediated changes in intracellular Aβ levels were different in primary astrocytes and 293sw cells. In conclusion, GCA and BFA inhibited DAPG-mediated sAPPα production in an ADAM10 inhibition-like manner and prevented DAPG-mediated sAPPα and Aβ secretion. Additionally, sAPPα and Aβ intracellular trafficking or secretory pathways were cell-type-dependent.

## 4. Discussion

**Figure 11:**
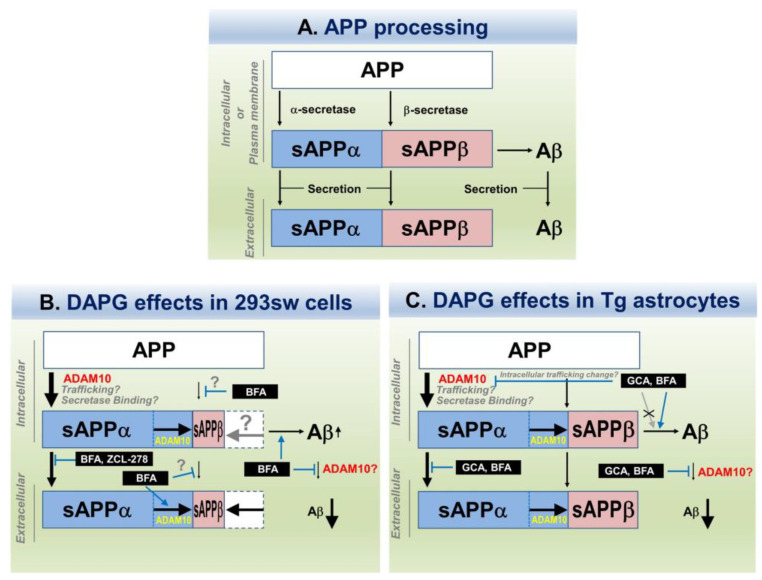
Schematic illustration of the mechanisms activated by DAPG to decrease Aβ levels and increase sAPPα levels in 293sw cells and primary astrocytes from APPsw/PS1dE9 transgenic mice. (**A**) APP processing pathways leading to Aβ and sAPPα production and secretion. (**B**) Effects of DAPG on Aβ and sAPPα production and secretion in 293sw cells and impact of the trafficking inhibitors BFA and ZCL-278. (**C**) DAPG effects on Aβ and sAPPα production and secretion in primary astrocytes from APPsw/PS1dE9 transgenic mice and impact of the trafficking inhibitors GCA and BFA.

The present study shows that DAPG, a low-molecular-weight phloroglucinol derivative, decreased Aβ secretion in AD cellular and animal models and might be a drug candidate for AD treatment. We screened the effects of various commercially available phloroglucinol derivatives of low molecular weight described in previous reports [12,13] on cell viability and Aβ levels (Figure 1) because many derivatives investigated so far are purified from natural sources and have large molecular weight, which are disadvantages for their use and availability as early drug candidates [24]. The chemical activity of DAPG, which was the most effective at lowering Aβ secretion, has rarely been reported in humans, and many reports on DAPG are restricted to its role in preventing plant diseases induced by soil-harmful bacteria, fungus, and protozoa [10]. A single study concerned eukaryotes and showed DAPG-mediated ROS formation, permeability of the plasma membrane, and changes in intracellular homeostasis in yeast [11]. However, there are no such data in humans. Although systemic DAPG treatment of an AD animal model failed to prevent Aβ accumulation and memory decline, possibly because of the BBB impermeability predicted by computational approach, the present study suggests that DAPG is a novel AD drug candidate that reduces Aβ secretion through novel mechanisms such as regulation of ADAM10 and intracellular trafficking (Table 2 and Table 3, and Figure 11). In addition to the reducing effect of Aβ secretion, according to previous reports, polyhydroxyl phenolic compounds such as DAPG can inhibit amyloid aggregation (oligomerization and fibrilization) [25,26]. Although it remains to be further demonstrated whether DAPG inhibits Aβ aggregation, these reports suggest that DAPG may have multi-mode of function to inhibit AD pathogenesis and can be an important drug candidate for AD.

To investigate the mechanisms of DAPG-mediated reduction in Aβ secretion, we initially analyzed changes of Aβ production, degradation, and clearance (Figure 3). DAPG treatment did not clear exogenous Aβ intracellularly or extracellularly (Figure 3E,F). However, DAPG reduced Aβ production or secretion from the cells as shown in 293sw cells (Figure 3) as well as primary astrocytes (Figure 5) and hippocampi of APPsw/PSdE9 transgenic mice (Figure 6). Next, we identified DAPG-associated changes in APP processing, namely sAPPα production (intracellular sAPPα) and secretion (sAPPα in the medium), to be common to the above-mentioned models. Other changes in APP processing were specific to the cell type: DAPG treatment of 293sw cells reduced intracellular C83 and secreted sAPPβ levels, likely as a result of sAPPs reduction and sAPPα increase induced by DAPG (Figure 3C), whereas DAPG treatment in the hippocampus or primary astrocytes of APPsw/PS1dE9 mice increased C83 levels (Figure 5F and Figure 6F). Because it was well-reported that APP processing is different depending on the cell type [27,28], we concluded that intracellular sAPPα production and secretion were major events associated with DAPG-mediated Aβ reduction. Therefore, the mechanisms activated by DAPG to induce sAPPα production and secretion were examined.

First, we confirmed that the DAPG-mediated production of sAPPα occurred in intracellular organelles. In fact, α-secretase is mainly located in the plasma membrane rather than in other intracellular microorganelles [21,22], but some reports have suggested that the intracellular activity of α-secretases such as ADAM10 is crucial for cleaving APP [29,30]. By examining sAPPα intracellular location, we confirmed that DAPG increased intracellular sAPPα levels in organelle-enriched fractions, particularly in the ER, lysosomes, and cis- and trans-Golgi networks, but not in endosomes, as previously reported (Figure 4) [30]. Although it is not known yet whether sAPPα is produced or accumulates in these organelles, the present study shows that intracellular sAPPα production was strongly associated with DAPG-mediated Aβ reduction.

Our data also revealed that ADAM10, but not ADAM17, was affected by DAPG and increased sAPPα production and secretion induced by DAPG in 293sw cells and transgenic primary astrocytes (Figure 7B and Figure 8C). However, DAPG-mediated reduction in secreted Aβ levels was not prevented by the ADAM10 inhibitor, GI294023X (Figure 7D and Figure 8E). According to previous reports, α-secretase can compete with β-secretase and modulate the balance between the non-amyloidogenic and amyloidogenic pathways [22,31]. This hypothesis seemed to apply well in our cellular system (293sw and transgenic primary astrocytes) in the absence of DAPG treatment (Figure 7C and Figure 8D). Next, if the above hypothesis is correct, since GI294023X may affect intracellular change of Aβ, not its secretion, we investigated intracellular change of Aβ by GI294023X in DAPG-treated cells. Intracellular Aβ levels were increased by GI294023X and DAPG co-treatment compared with those observed after DAPG treatment (Figure 7E and Figure 8G), indicating that ADAM10 participated in DAPG-mediated Aβ reduction within the cells, whereas Aβ secretion by DAPG might be blocked by ADAM10 inhibition or might be independent of ADAM10 activity. In addition, the effects of α-secretase inhibition in basal conditions, without DAPG, were different in 293sw cells from those in transgenic primary astrocytes. GI294023X treatment reduced basal sAPPα secretion in 293sw cells but not in astrocytes (Figure 7B and Figure 8B), whereas it increased secreted Aβ levels in both cell models (Figure 7C and Figure 8D). These different effects of GI294023X with or without DAPG between cell types indicated that the constitutive activity or subtypes (ADAM10 or ADAM17) of α-secretase depended on the cell type, but DAPG modulated ADAM10 α-secretase activity similarly in both cell types. Indeed, constitutive α-secretase cleaves APP and other substrate proteins and certain stimuli such as phorbol 12-myristate, 13-acetate (PMA) can increase the cleavage by modulating the activity, transcription, or location of α-secretase [22,32]. Therefore, DAPG might regulate ADAM10 activity despite different constitutive activities is various cell types. Moreover, because α-secretase total activity in cells was not changed by DAPG in the present study (Figure S3), responses of α-secretase to DAPG in both cell types seem to be regulated by changes in intracellular localization or binding partners of α-secretase.

In addition to the role of ADAM10, the present study suggests an implication of other mechanisms regulating Aβ secretion affected by DAPG to reduce Aβ levels. Indeed, although DAPG and GI294023X co-treatment increased intracellular Aβ levels, DAPG alone did not provoke a decrease in intracellular Aβ levels but rather an increase in 293sw cells or no change in the primary astrocytes, indicating that ADAM10 only partly participated in the response to DAPG and further mechanisms affected Aβ secretion. Therefore, we modulated the intracellular trafficking leading to protein secretion using various inhibitors, i.e., GCA and BFA [33], CI-976 [34], ZCL-278 [35], vacuolin-1 [36], EHNA [37], and retro-2 [38]. ZCL-278, an inhibitor of CDC42 mainly located in the Golgi complex, blocked the most effectively DAPG-mediated changes of sAPPα levels in 293sw cells and had similar effect than those of the ADAM10 inhibitor (Figure S4). However, ZCL-278 did not reverse DAPG-mediated reduction in Aβ levels in the culture medium, whereas BFA attenuated this reduction and increased Aβ intracellular levels and secretion (Figure 9G,H). These differential effects of ZCL-278 and BFA indicated that DAPG-mediated changes of sAPPα and Aβ levels were at least partly independent. BFA inhibits ADP-ribosylation factors–guanine–nucleotide–exchange factor (ARF-GEFs) including Golgi-specific brefeldin-resistance factor a (GBF1) and BFA-inhibited guanine nucleotide exchange protein 1 and 2 (BIG1 and BIG2) [39,40,41]. Compared with BFA, GCA is a relatively specific inhibitor of GBF1 but not BIG1/2 [33]. Moreover, GBF1, a target of BFA and GCA, is located in the cis-Golgi complex, and BIG1 and BIG2, targets of BFA, are located in trans-Golgi complex and trans-Golgi network (TGN), respectively. This differential localization likely results in the distinct roles of the ARF-GEFs and their inhibitors [33,40]. Furthermore, BIG2 is also associated with recycling endosomes, an organelle other than the Golgi complex [39,41], and BFA also disrupts sorting pathways in the endosomal system [39,42]. These reports suggest that BFA effects in DAPG-treated 293sw cells depended on BIG1, BIG2, or endosomal trafficking, but not on GBF1 or the cis-Golgi compartment. In contrast, in transgenic primary astrocytes, both GCA and BFA reversed DAPG-mediated effects on sAPPα and Aβ processing and secretion (Figure 10), possibly indicating that GBF1-dependent effects in the Golgi complex are commonly regulated by GCA and BFA. Finally, sAPPα levels after co-treatment with DAPG and GCA or BFA of the primary astrocytes resembled to those observed after cell exposure to the ADAM10 inhibitor in the astrocytes and 293sw cells or ZCL-278 in 293sw cells, but Aβ levels induced by the co-treatment with ZCL-278 or GCA and BFA were different, which also suggested that sAPPα and Aβ production machineries were independent and specific from the cell type. More detail mechanisms of DAPG action need to be elucidated, including whether DAPG affects several independent pathways to regulate sAPPα and Aβ levels or a key signaling mediator regulating intracellular trafficking in a cell-type-dependent manner.

Altogether, the present study suggests that DAPG is a novel drug candidate for AD treatment modulating ADAM10 and intracellular trafficking in cellular and transgenic animal models of AD. Although DAPG was predicted not to cross the BBB and its exact molecular targets remain unknown, this study identified a novel mechanism to reduce Aβ secretion and confirmed that DAPG might be an important compound in the development of novel AD therapeutics, providing that chemical modifications of DAPG allowing it to cross the BBB and improve the memory decline are identified.

## Figures and Tables

**Table 1:** Permeability prediction of phloroglucinol derivatives using LogP (distribution coefficient P) value. Values mean lipid bilayer permeability (0 < LogP < 3, optimal; LogP < 0, poor lipid bilayer permeability; LogP > 3, poor aqueous solubility).

Compound	LogP Value	Meaning
Phloroglucinol	0.803	Optimal
1,3,5-trimethylbenzene	2.612	Optimal
Scyllo-inositol	−3.835	Poor lipid bilayer permeability
Flopropione	1.396	Optimal
2,4,6-Trihydroxybenzaldehyde	0.616	Optimal
Maclurin	1.446	Optimal
Phloroacetophenone	1.006	Optimal
2,4-diacetylphloroglucinol	1.209	Optimal
2,4,6-triacetylphloroglucinol	1.411	Optimal
Phloroglucinol triacetate	1.462	Optimal
2,4,6-trihydroxybenzoic acid	0.502	Optimal
Phloretin	2.325	Optimal

**Table 2:** Summary of changes in Aβ parameters by GI293023X, BFA, or GCA alone and co-treatment with DAPG. Changes are shown compared to untreated cells (untreated) and DAPG-treated cells (DAPG-treated), respectively (Ic, intracellular; Ec, extracellular; n.d., not determined).

Compound	Inhibitory Target	Comparison Target	Molecular Actions					
			Secreted Aβ		Intracellular Aβ		Ratio of Ic Aβ per Ec Aβ	
			293sw	Primary Astrocytes	293sw	Primary Astrocytes	293sw	Primary Astrocytes
**GI294023X**	**ADAM10**	Untreated	↑	↑	−	−	n.d.	n.d.
		DAPG-treated	−	−	↑	↑	n.d.	n.d.
**BFA**	GBF1/BIG1/BIG2	Untreated	↓	↓	↓	−	n.d.	n.d.
		DAPG-treated	↑	↑	↑	↑	↓	↓
**GCA**	GBF1	Untreated	↓	↓	↓	↓	n.d.	n.d.
		DAPG-treated	−	↑	−	−	−	↓

**Table 3:** Summary of changes in sAPPs parameters by GI293023X, BFA, or GCA alone and co-treatment with DAPG. Changes are shown compared to untreated cells (untreated) and DAPG-treated cells (DAPG-treated), respectively (Ic, intracellular; Ec, extracellular; n.d., not determined).

Compound	Inhibitory Target	Comparison Target	Molecular Actions									
			sAPPα (Cells)		sAPPα (Media)		sAPPs (Media)		sAPPα/sAPPs (Media)		Ratio of Ic sAPPα/Ec sAPPα	
			293sw	Primary Astrocytes	293sw	Primary Astrocytes	293sw	Primary Astrocytes	293sw	Primary Astrocytes	293sw	Primary Astrocytes
**GI294023X**	**ADAM10**	Untreated	−	↓	−	−	−	−	↓	−	n.d.	n.d.
		DAPG-treated	↓	↓	↓	↓	−	−	↓	↓	n.d.	n.d.
**BFA**	GBF1/BIG1/BIG2	Untreated	−	↑	↓	↑	↓	↓	−	↑	n.d.	n.d.
		DAPG-treated	−	↓	↑	↓	↑	−	−	↓	↓	↓
**GCA**	GBF1	Untreated	−	−	−	↓	↓	↓	−	↑	n.d.	n.d.
		DAPG-treated	−	↓	−	−	−	−	−	↓	↓	↓

## Data Availability

All data generated or analyzed during this study are available from the corresponding author upon reasonable request.

## Supplementary Materials

The following are available online at https://www.mdpi.com/article/10.3390/cells11162585/s1, Figure S1: Examination of DAPG effects on spatial memory and APP processing in APPsw/PS1dE9 transgenic mice and computational prediction of brain–blood barrier permeability to DAPG, Figure S2: Regulation of DAPG-mediated changes in sAPPα and sAPPs levels by β- and γ-secretase inhibitors in 293sw cells, Figure S3: Examination of DAPG-mediated changes in α-secretase activity, Figure S4: Examination of the effects of other intracellular trafficking inhibitors on DAPG-mediated Aβ level decrease and sAPPα level increase in 293sw cells, and Table S1: Antibodies used for this study.
