# Supplementary material for: 2,4-Diacetylphloroglucinol Reduces Beta-Amyloid Production and Secretion by Regulating ADAM10 and Intracellular Trafficking in Cellular and Animal Models of Alzheimer’s Disease
Source: Cells. 2022 Aug 19;11(16):2585. doi: 10.3390/cells11162585 (PMC9406471; doi:10.3390/cells11162585)
Supplement: Supplementary file 1 [file cells-11-02585-s001.zip › cells-1861191-supplementary.pdf]

Supplementary materials

BACE inhibitor IV (BACEi IV), a β-secretase inhibitor, was purchased from Calbiochem (USA). DAPT, a γ-secretase inhibitor, EHNA hydrochloride, and retro-2 were purchased from SIGMA. ZCL-278, CI-976, and vacuolin-1 were obtained from CAYMAN chemicals (USA).

Supplementary methods

Morris water maze test

Spatial learning of 8-month-old transgenic mice was examined using the water maze test with hidden platform. The maze pool was circular with a diameter of 150 cm and a height of 40 cm. The circular platform (10 cm diameter) was hidden 1.5 cm below the surface of the water made opaque with non-toxic white color paint. The pool was located in a soundproof test room, which contained various visual cues including a different poster on each wall. The test consisted of 3–4 trials per day with intertrial intervals of 15 min spent on the platform. The location of the starting point varied daily. The time spent swimming and navigated swim path were recorded by an EthoVision automated video tracking system (Noldus information technology, Netherlands). If a mouse did not find the platform after 120 s, it was placed on the platform. On the fifth day, a probe test, in which the platform was removed from the pool, was performed after the trials. The total distance swum during a period of 120 s was recorded and mean target quadrant occupancy was calculated by dividing the distance swum in the platform quadrant by the total distance swum.

Measurement of α-secretase activity

The activity of α-secretase was measured in the TME fraction using an α-secretase-specific fluorogenic substrate as reported previously with modifications [14]. Briefly, 2×10^6 cells were homogenized on ice in extraction buffer (10 mM Tris-HCl [pH7.4], 1 mM MgCl2, 2 mM EGTA) containing a protease inhibitor cocktail for 10 min. Then, the homogenate was passed 10 times through a 26-gauge needle and centrifuged at 1,000 × g. The supernatant was centrifuged again at 100,000 x g for 1 h in an ultracentrifuge with a TLA-100.2 rotor (Beckman, USA). The

resultant pellet, which was the TME fraction, was resuspended in a buffer containing 10 mM Tris-HCl (pH 7.4), 1 mM $MgCl_2$, 2 mM EGTA, 0.5% Triton X-100, 1% CHAPSO (Sigma, USA), and a protease inhibitor. Total protein concentration was measured using the BCA method and 1–2 μg of membrane proteins were transferred in a well of an opaque 96-well plate. An α-secretase fluorogenic substrate was added (10 μM; Calbiochem, USA) and incubated at 37°C for 30–60 min in α-reaction buffer (20 mM Tris [pH 8.0]). After the reaction termination, fluorescence was measured at 350 nm/490 nm (excitation/emission) using an Infinite M200 PRO microplate reader. Alternatively, to measure the direct relationship between DAPG and α-secretase, various doses of DAPG were added to 10 μg of total membrane proteins from untreated control cells and the mixture was incubated at 37℃ for 1 h before being analyzed as described above.

Preparation of the endoplasmic reticulum (ER)-enriched (ERE) fraction

ER proteins were isolated using the Minute ER enrichment kit (Invent, USA) following the manufacturer's instructions. Briefly, 2×10^6 cells were homogenized on ice in buffer A containing a protease inhibitor cocktail for 30 s and placed in a filter cartridge. After centrifugation at 16,000 × g, the pellet was resuspended and centrifuged at 2,000 × g to remove the nuclear fraction and non-lysed cells. The supernatant was centrifuged again at 8,000 × g and the resultant supernatant was mixed with 1/10 volume of buffer B. After incubation at 4℃ for 30 min, the sample was centrifuged at 8,000 × g for 10 min. The pellet was resuspended in buffer A and 1/10 volume of buffer C was added. After incubation at room temperature for 10 min, the mixture was centrifuged at 8,000 × g for 5 min. An equal volume of buffer D was added to the supernatant and the solution was incubated at −70℃ for 20 min. After centrifugation at 10,000 × g for 10 min, the final pellet, which was the ERE fraction, was stored at −70℃.

Preparation of the lysosome-enriched (LyE) fraction

Lysosomal proteins were isolated using the Minute lysosome isolation kit for mammalian cells/tissues (Invent, USA) following the manufacturer's instructions. Briefly, 2×10^6 cells were homogenized on ice in buffer A containing a protease inhibitor cocktail for 10 min and placed in a filter cartridge. After centrifugation at 16,000 × g, the pellet was resuspended and centrifuged at 2,000 × g to remove the nuclear fraction and non-lysed cells. The supernatant was centrifuged again at 11,000 × g for 15 min and the resultant pellet contained mitochondria. The supernatant was centrifuged at 16,000 × g for 30 min and the pellet was resuspended with ice col buffer A. After centrifugation at

2,000 × g for 4 min, the supernatant was mixed with 1/2 volume of buffer B and incubated on ice for 30 min. After a final centrifugation at 11,000 × g for 10 min, the pellet, which was the lysosome-enriched (LyE) fraction, was stored at −70°C.

Preparation of the endosome-enriched (EnE) fraction

Endosomal proteins were isolated using the Minute endosome isolation and cell fractionation kit (Invent, USA) following the manufacturer's instructions. Briefly, 2 × 10^6 cells were homogenized on ice in buffer A containing a protease inhibitor cocktail for 10 min and placed in a filter cartridge. After centrifugation at 16,000 × g, the pellet was resuspended and centrifuged at 700 × g to remove the nuclear fraction and non-lysed cells. The supernatant was centrifuged again at 16,000 × g for 60 min and the resultant supernatant was mixed with 1/2 volume of buffer B. After incubation for 2 h at 4°C, samples were centrifuged at 10,000 × g for 30 min and the pellet, which constituted the EnE fraction, was stored at −70°C.

Preparation of Golgi complex-enriched fractions

Proteins from the Golgi apparatus and secretory vesicles (cis-Golgi and trans-Golgi-enriched [cGE and tGE, respectively] fractions, respectively) were isolated using the Minute Golgi apparatus enrichment kit (Invent, USA) following the manufacturer's instructions. Briefly, 2 × 10^6 cells were homogenized in buffer A containing a protease inhibitor cocktail and placed in a filter cartridge. After centrifugation at 16,000 × g for 30 min, the supernatant was mixed with an equal volume of buffer B and incubated on ice for 15 min. After centrifugation at 8,000 × g for 6 min, the supernatant containing secretory vesicles of the trans-Golgi apparatus was concentrated by incubating it on ice with 1/8 volume of buffer D for 15 min and centrifugating it at 16,000 × g for 30 min. The resultant pellet was incubated 20 min on ice after resuspension with 1/2 volume of buffer C and centrifugated at 8,000 × g for 10 min. The final pellet constituted the cGE fraction. Both cGE and tGE fractions were stored at −70°C.

Supplementary figures

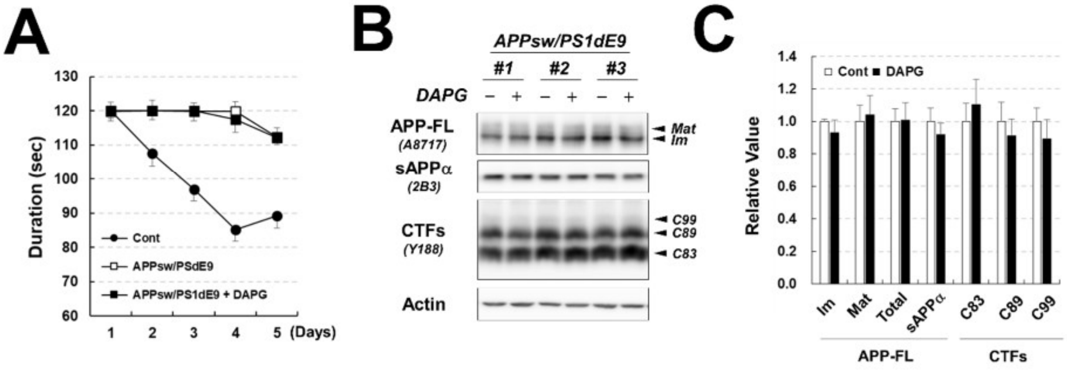

Figure S1. Examination of DAPG effects on spatial memory and APP processing in APPsw/PS1dE9 transgenic mice and computational prediction of brain–blood barrier permeability to DAPG. (**A**) Spatial memory analysis using the Morris water maze test after DAPG intraperitoneal injection (20 mg/kg, daily injection for 9 days) of APPsw/PS1dE9 transgenic mice. The test was conducted the last 5 days of the injection series. (**B, C**) Immunoblot analysis of APP processing in hippocampi of the control and DAPG-injected transgenic mice (**B**) and densitometry analysis of the immunoblots (**C**). Values in the graphs are presented as means ± SEMs (n = 8).

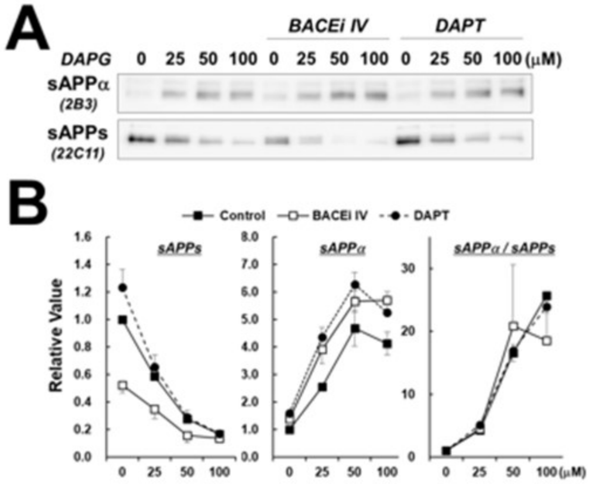

Figure S2. Regulation of DAPG-mediated changes in sAPPα and sAPPs levels by β- and γ-secretase inhibitors in 293sw cells. (**A**) Immunoblot analysis of sAPPα and sAPPs in 293sw cells treated with 0–100 μM DAPG and 10 μM BACE inhibitor IV (β-secretase inhibitor) or 10 μM DAPT (γ-secretase inhibitor) for 8 h. (**B**) Densitometry analysis of sAPPα and sAPPs levels in the medium and the ratio of sAPPα to sAPPs levels on the immunoblots presented in (**A**). Values are presented as means ± SEMs (n = 3).

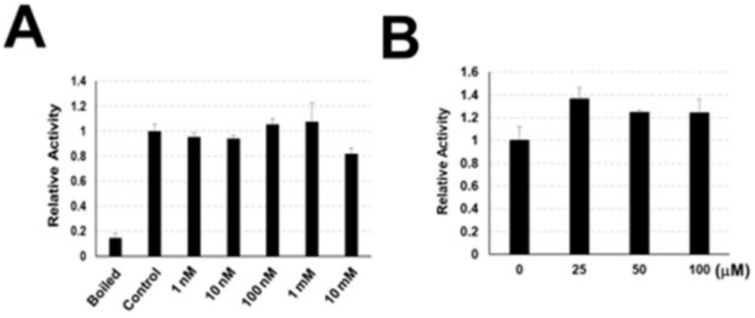

Figure S3. Examination of DAPG-mediated changes in α-secretase activity. (**A**) Analysis of α-secretase activity in the membrane fraction of 293sw cells treated with the indicated doses of DAPG for 1 h. The values were normalized to the activity in untreated 293sw cells and are presented as means ± SEMs (n = 4). (**B**) Analysis of α-secretase activity in 293sw cells treated with 0–100 μM DAPG for 8 h. Values are presented as means ± SEMs (n = 3).

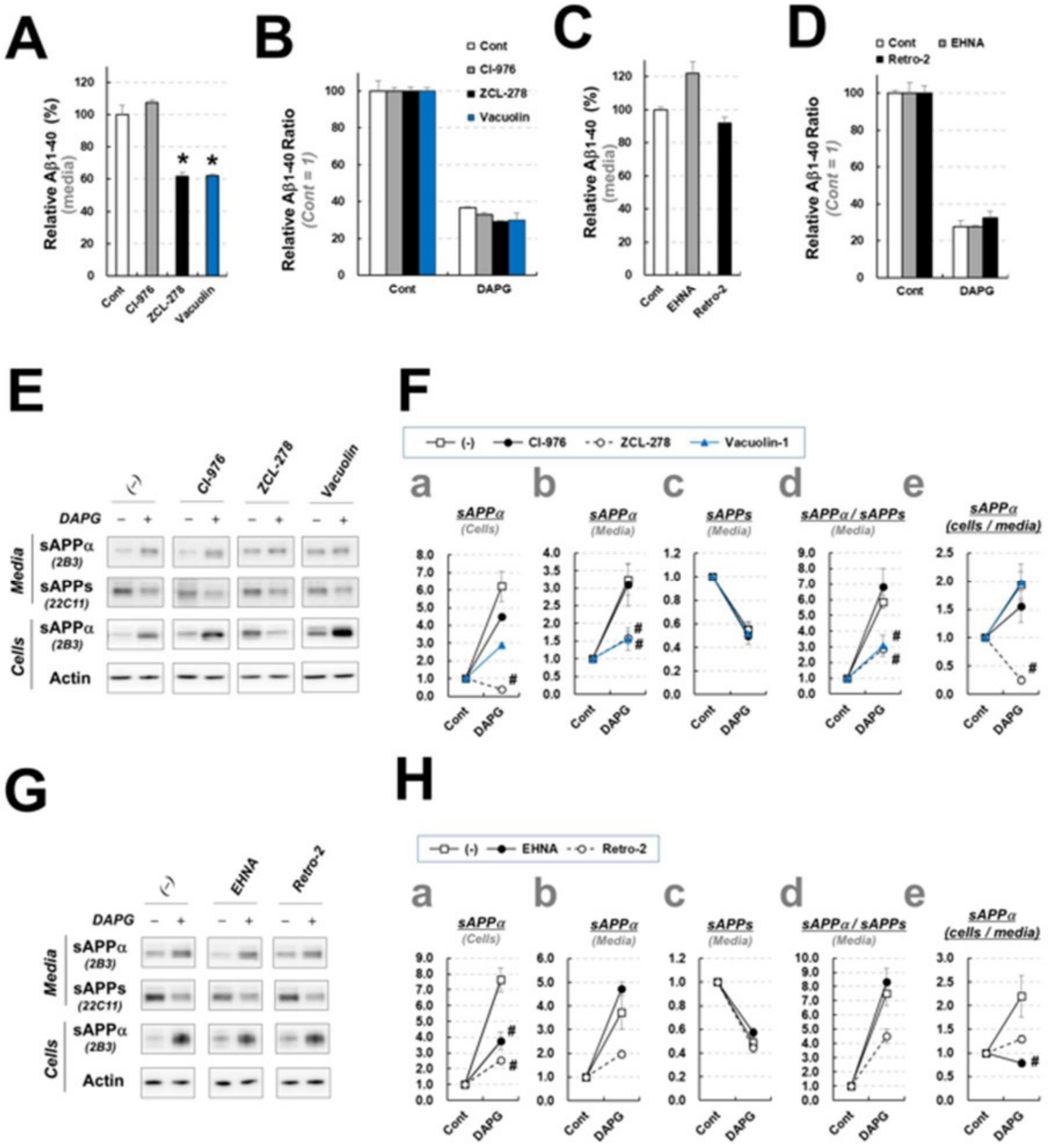

Figure S4. Examination of the effects of other intracellular trafficking inhibitors on DAPG-mediated Aβ level decrease and sAPPα level increase in 293sw cells. (**A**) ELISA analysis of $A\beta_{1\text{-}40}$ content in the culture medium of 293sw cells treated with CI-976 (20 μM), ZCL-278 (50 μM), or vacuolin-1 (5 μM) for 8 h. (**B**) Relative $A\beta_{1\text{-}40}$ content in the medium of 293sw cells treated with 50 μM DAPG with or without CI-976, ZCL-278, and vacuolin-1 for 8 h obtained by ELISA. (**C**) ELISA analysis of $A\beta_{1\text{-}40}$ levels in the culture medium of 293sw cells treated with EHNA (10 μM) or retro-2 (5 μM) for 8 h. (**D**) Relative $A\beta_{1\text{-}40}$ content in the medium of 293sw cells treated with 50 μM DAPG with or without EHNA or retro-2 for 8 h obtained by ELISA. (**E**) Immunoblot analysis of sAPPα and sAPPs levels in 293sw cells treated with DAPG and CI-976, ZCL-278, or vacuolin-1 for 8 h. (**F**) Densitometry analysis of sAPPα and sAPPs levels in the medium or cells from immunoblots presented in (**E**). This panel shows the relative ratio between controls and cells treated with DAPG alone (untreated) or with DAPG and CI-976, ZCL-278, or vacuolin-1. The levels of sAPPα in cells (**a**) and the medium (**b**) and sAPPs in the medium (**c**) as well as the ratios of sAPPα to sAPPs levels in the medium (**d**) and of sAPPα levels in cells to those in the medium (**e**) are

presented. (**G**) Immunoblot analyses of sAPPα and sAPPs levels in 293sw cells treated with DAPG and EHNA or retro-2 for 8 h. (**H**) Densitometry analysis of sAPPα and sAPPs levels in the medium or cells from immunoblots presented in (**G**). The ratios between controls and cells treated with DAPG alone (untreated) or DAPG with EHNA or retro-2 are shown. The levels of sAPPα in cells (**a**) and the medium (**b**) and sAPPs in the medium (**c**) as well as the ratios of sAPPα to sAPPs levels in the medium (**d**) and of sAPPα levels in cells to those in the medium (**e**) are shown. All values in graphs are presented as means ± SEMs (n = 3). Statistical significances of differences between control untreated and treated groups in panel **A** were determined using Student's t-test (*$p < 0.05$). In panels **F** and **H**, statistical significances of differences between DAPG-treated and DAPG- and the trafficking inhibitor-treated groups ($^{\#}p < 0.05$) were determined using one-way ANOVA followed by Tukey's test.

Supplementary table

Table S1. Antibodies used for this study

Antigen (Clone Name)	Host	Clonality	Dilution	Company	Cat. No.
C-term of APP (A8717)	Rabbit	Polyclonal	1 : 2,000	Sigma, USA	A8717
C-term of APP (Y188)	Rabbit	Monoclonal	1 : 2,000	Abcam, UK	ab32136
N-term of APP (22C11)	Mouse	Monoclonal	1 : 1,000	Merck Millipore, USA	MAB538
sAPPα (2B3)	Mouse	Monoclonal	1 : 150	IBL, Japan	11088
ADAM10	Rabbit	Monoclonal	1 : 1,000	Abcam, UK	ab124695
ADAM17	Rabbit	Monoclonal	1 : 1,000	Abcam, UK	ab2051
Na/K-ATPase α1	Mouse	Monoclonal	1 : 1,000	Santa Cruz, USA	sc-21712
TGN46	Rabbit	Polyclonal	1 : 500	Novus, USA	NBP1-62556
EEA1	Rabbit	Monoclonal	1 : 1,000	Cell Signaling Technology, USA	3288
α-tubulin	Mouse	Monoclonal	1 : 3,000	Sigma, USA	T6199
β-actin	Mouse	Monoclonal	1 : 5,000	Sigma, USA	A5316
